# Pilot-scale ultraviolet-C and nano-second pulsed electric field processing of milk: Microbial inactivation and preservation of serum proteins

**DOI:** 10.1016/j.crfs.2026.101546

**Published:** 2026-08-26

**Authors:** Siwei Li, Sjef Boeren, Betty.C.A.M. van Esch, Kasper A. Hettinga

**Affiliations:** a Dairy Science and Technology, Food Quality & Design Group, Wageningen University & Research, Wageningen, the Netherlands; b Laboratory of Biochemistry, Wageningen University & Research, Wageningen, the Netherlands; c Division of Pharmacology, Utrecht Institute for Pharmaceutical Sciences, Faculty of, Science, Utrecht University, Utrecht, the Netherlands

**Keywords:** UVC, Nano-second PEF, Microbial reduction, Milk serum proteins, Milk serum proteome

## Abstract

Non-thermal processing methods are increasingly used as alternatives to heat treatment for milk due to their potential to preserve heat-sensitive components. In this study, pilot-scale ultraviolet-C (UVC) and nano-second pulsed electric field (nanoPEF) equipment, as opposed to lab-scale instruments, were used to treat milk. The effects of these two non-thermal processing methods on microbial reduction and milk serum proteins were evaluated. Milk samples were treated by UVC irradiation (0.384–4.8 kJ/L), nanoPEF (29–182 kJ/L), and high-intensity pasteurization (85 °C, 5 min), followed by milk serum preparation. The profile of native serum proteins was analyzed using DUMAS, SDS-PAGE, and label-free LC-MS/MS-based proteomics. Structural changes and oxidative modifications were assessed via measurement of the protein's surface hydrophobicity, number of thiol groups, available free amino and carbonyl groups, and N-formyl kynurenine levels. At UVC doses above 1.44 kJ/L, and nanoPEF levels above 29 kJ/L combined with mild heating at 50 °C, reductions in the measured microbial indicator groups, including aerobic mesophilic bacteria, thermoduric bacteria, and coliforms, were comparable to those observed after a high-intensity pasteurization treatment in this study. Meanwhile, UVC and nanoPEF preserved over 88% and 95% of milk serum proteins, respectively, versus 56% in heat-treated milk. Minimal structural changes and oxidation levels in non-thermal treated milk serum proteins indicated better preservation compared to heating, closely resembling raw milk. Further pathogen challenge studies are required to validate microbial safety under industrially relevant conditions. Conclusively, these two non-thermal techniques show potential for dairy thermal processing, maintaining product characteristics closer to raw milk.

## Highlights

• Pilot-scale UVC and nanoPEF at varying intensities were applied to milk processing.
• Both treatments achieved microbial inactivation comparable to pasteurization.
• UVC and nanoPEF preserved >88% and >95% of milk serum proteins, respectively.
• Structural and oxidative changes in non-thermal treated milk proteins were minimal.
• Milk quality after UVC and nanoPEF processing closely resembled that of raw milk.

## Graphical abstract

**Figure:**
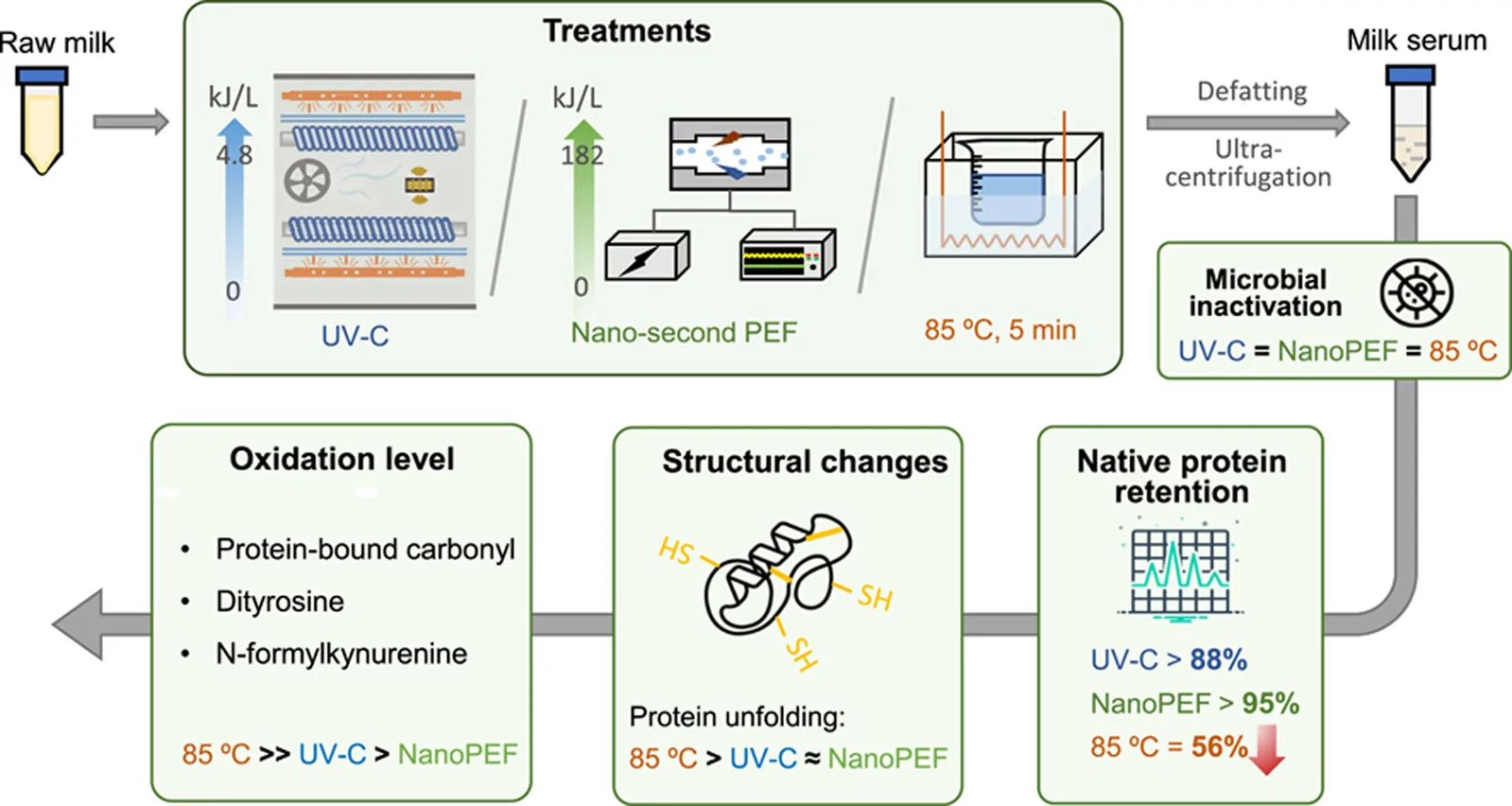

## 1 Introduction

Milk is one of the most popular protein and micronutrient sources in the human diet, with a high nutritional value (Boland and Hill, 2019). Bovine milk consists of all the essential nutrients required for the infants' growth and development, and provides a range of bioactive components that play an important role in brain and immune system development (Lin et al., 2021). Among these bioactive components, the milk serum proteins provide various biological functions (Hettinga et al., 2011).

Raw milk exerts anti-allergic and bacteriostatic abilities due to various allergy-protective and antibacterial milk serum proteins and peptides (Sozańska, 2019). Epidemiological studies reported the association between raw farm milk consumption and protection against allergic diseases (Perkin and Strachan, 2006; Waser et al., 2007). Although raw milk consumption can theoretically be a way to prevent allergies, it is discouraged by governmental agencies because of the possible presence of pathogens (Brick et al., 2020). Thus, heat treatment is applied to milk and dairy products to ensure their microbiological safety and shelf life. However, these bioactive milk serum proteins are heat-sensitive and therefore susceptible to damage during heat processing. Thermal processes can lead to immunologically-active protein denaturation, aggregation and glycation. Consequently, the allergy-protective effects and bacteriostatic capacity of raw milk are lost (Brick et al., 2017; Loss et al., 2011). Several studies indicated the effect of heat treatment on immunologically-active proteins of both bovine and human milk. It was reported that approximately 20%, 45% and 50% of milk serum proteins were lost after low temperature pasteurization (20s at 72 °C), high temperature pasteurization (23s at 93 °C), UHT treatment (142 °C for 5 s) and boiling (30s at 100 °C), respectively (Brick et al., 2017). Holder pasteurization (HoP, the most common process applied to donor human milk) at 63 °C for 30 min was shown to denature 50% of lactoferrin and 20% of IgG (Liu et al., 2020a, Liu et al., 2020b).

In contrast to thermal treatments that lead to a certain loss of immune-active proteins, non-thermal treatments are gaining increasing attention in the dairy industry. Non-thermal processing refers to food preservation methods that eliminate the use of high temperatures to preserve nutritional quality. These novel technologies enable environmentally friendly and sustainable food processing with reduced energy and water consumption (Singh and Huppertz, 2020). Their potential for dairy processing is associated with microbial safety, preservation of nutritional and functional components, improved process efficiency, and industrial applicability (Digambar Sawale et al., 2024). Thus, they may play an important potential role in microbial inactivation while simultaneously protecting heat-sensitive proteins. Ultraviolet-C (UVC) and nano-second pulsed electric field (nanoPEF) are two emerging technologies for food preservation. Compared with UVA and UVB, UVC has a shorter wavelength (200-280 nm) and stronger microbicidal efficacy. It's able to destroy bacteria, moulds, viruses, yeasts, protozoa, and algae by causing photoproducts of DNA bases, which makes the DNA strands unable to replicate, thus leading to cell death (Christen et al., 2013). NanoPEF enables the permeabilization of inner and outer cell membranes and the activation of ionic channels. This leads to an incremental increase in cytoplasmic Ca^2+^ concentration. The resulting signaling cascade produces diverse cellular outcomes, ranging from apoptosis to differentiation and proliferation (Ruiz-Fernández et al., 2022). Compared with microsecond PEF, nanosecond PEF applies ultrashort (1-100 ns) and high-intensity electric fields that irreversibly induce intracellular damage and nanoporation of cellular membranes. It enables the effective inactivation of more resistant microorganisms, such as bacterial spores, yeasts, and certain viruses (Novickij et al., 2018). With a lower temperature increase, recent studies have shown that UVC and nanosecond PEF treatments can better retain heat-sensitive bioactive milk proteins than conventional heat treatment. For example, UV-C processing retained approximately 80% of native lactoferrin and immunoglobulins in raw milk (Y. Liu et al., 2024). Nanosecond PEF-treated human milk showed over 60% retention of lysozyme, lactoperoxidase, and lactoferrin and complete retention of IgA and xanthine oxidase compared with raw milk (J. Zhang et al., 2023). Despite the advantages of UV and PEF in preserving milk quality, the use of excessively intense non-thermal treatment conditions may induce alterations to the native secondary and tertiary structures of milk serum proteins, subsequently leading to protein denaturation, cross-linking, and aggregation (Hu et al., 2024; Zhao et al., 2020), similar to heat treatment.

Previous studies have demonstrated the feasibility of continuous-flow or pilot-scale UVC and PEF processing of milk, but have mainly focused on microbial inactivation and reactor or process performance (Chawla et al., 2021; Lee et al., 2015; Makararpong et al., 2024; Vashisht et al., 2024; J. Zhang et al., 2023). Proteomic studies have also shown that thermal and non-thermal processing can alter the milk serum proteome (Y. Liu et al., 2020). However, studies translating UVC and nanoPEF from laboratory-scale observations to pilot-scale milk processing while simultaneously linking microbial performance with proteome-wide protein retention, structural alterations, and oxidative changes remain limited. This gap is particularly relevant for the direct comparison of UVC and nanoPEF across different treatment intensities.

We hypothesize that under UVC and nanoPEF conditions achieving microbial reductions comparable to the high-intensity thermal pasteurization reference used in this study, the structure and profile of milk serum proteins will more closely resemble those of raw milk. This study thereby aimed to evaluate, at pilot plant scale, the effects of different intensities of UVC and nanoPEF with high-intensity pasteurization on naturally occurring microbes, milk serum proteome composition, and protein structure and oxidation. To achieve these aims, milk was treated by various intensities of UVC, nanoPEF and heating, after which the total microbial counts were determined by plate counting. Milk serum was prepared by acidification and centrifugation, and the changes in native milk serum protein concentrations were compared. The detailed milk serum protein changes were characterized by SDS-PAGE and LC-MS/MS. In addition, the structural changes were evaluated by milk serum surface hydrophobicity, thiol group and amino group contents. Furthermore, milk serum protein oxidation was measured by protein-bound carbonyl, N-formylkynurenine (NFK), and dityrosine formation.

## 2 Materials and methods

**Fig. 1:**
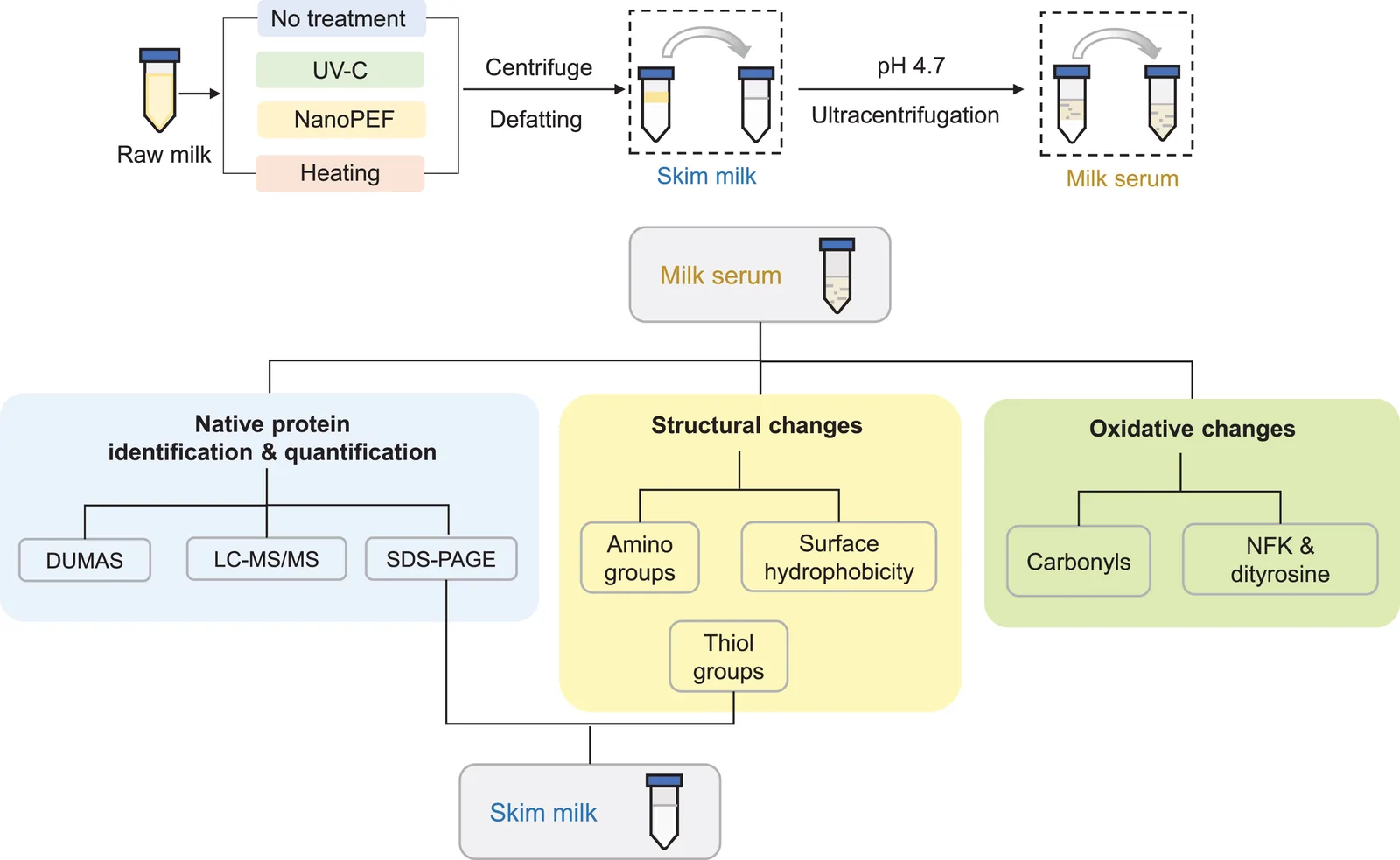
Schematic representation of the experimental design used.

The experimental design of this research is displayed in Fig. 1.

### 2.1 Materials and chemicals

The bovine milk samples used in this study were from two independent milk batches in the Netherlands: one batch for the UVC treatments and its corresponding untreated control, and one batch for the nanoPEF treatments and its corresponding untreated control. The raw fresh milk for UVC treatment was obtained through FrieslandCampina (Wageningen, The Netherlands) and processed at their pilot plant. The heat-treated sample was prepared from the same batch as the UVC-treated samples. The fresh raw milk used for nanoPEF treatment was sourced from a local dairy farm in Enschede by PEF Technologies and processed at their facility. All milk for the same treatment was collected and processed on the same day. Before processing, the milk was stored in tanks at 2-4 °C. After processing, samples intended for microbiological analysis and particle size measurements were stored at 4 °C and analyzed on the same day. The remaining samples were stored at −20 °C until further analysis.

NuPAGE 12% Bis-Tris protein gel, NuPAGE LDS sample buffer (4x), NuPAGE sample reducing agent (10x), NuPAGE MOPS SDS running buffer (20x) were purchased from Thermo Fisher Scientific (Massachusetts, USA). BlueRay pre-stained protein marker was purchased from Jena Bioscience (Jena, Germany). Coomassie Brilliant Blue R-250 Staining Solution was purchased from BIO-RAD (California, USA). Plate Count Milk Agar, violet Red Bile Agar, tris, dithiothreitol, urea, acrylamide, ammonium bicarbonate, formic acid, 2,2′-azino-bis(3-ethylbenzothiazoline-6-sulfonic acid, ABTS), hydrogen peroxide, trifluoroacetic acid, 5,5′-dithiobis-(2-nitrobenzoic acid) (DTNB), glycine, and 8-Anilinonaphthalene-1-sulfonic acid (ANS) were obtained from Merck (Darmstadt, Germany). Bovine Sequencing grade Trypsin (11047841001) was purchased from ROCHE (Basel, Switzerland).

### 2.2 Milk treatments

Four types of milk were used in this study: untreated milk, UVC-irradiated milk, nanoPEF-treated milk, and high-intensity pasteurized milk. For each treatment condition, three independent processing replicates were prepared from the same milk batch. The UVC and nanoPEF treatment intensities were selected to cover a range from mild to relatively high processing conditions within the operational capacity of the pilot-scale systems. The lower intensities were included to evaluate whether microbial reduction could be achieved while minimizing changes in milk serum proteins, whereas the higher intensities were selected to assess whether increased microbial reduction would be accompanied by greater protein loss, structural modification, or oxidation.

The high-intensity pasteurized milk was created by placing the raw whole milk into Greiner tubes (50 mL) and heating at 85 °C for 5 min, in a shaking water bath (200 rpm). This heating condition was selected to introduce clear and measurable heat-related changes in milk serum proteins, thereby providing a thermal reference for comparing the effects of different processing principles. In addition, this condition was chosen to provide substantial reductions in naturally occurring microbes, allowing comparison with UVC and nanoPEF treatments under the conditions tested in this study.

#### 2.2.1 Ultraviolet-C irradiation treatment

**Fig. 2:**
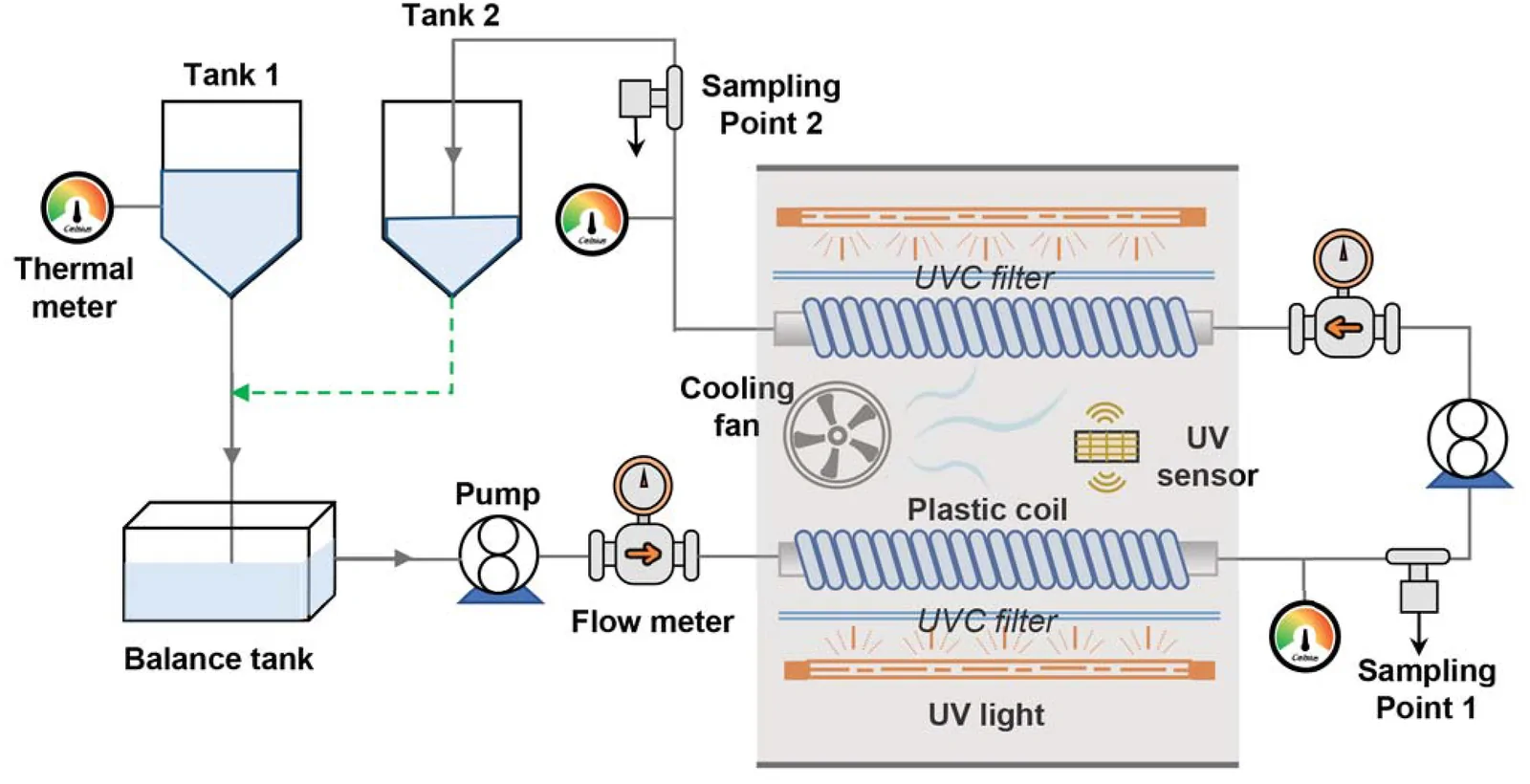
A schematic view of the UVC system. Tank 1 is the feed tank with raw milk, while tank 2 is the collection tank for UVC-treated milk. UV lamp, UVC filter, and plastic coils are placed in an opaque stainless-steel box (grey zone). The cooling fan keeps the milk at room temperature. The UV sensor is used to monitor and ensure the UV light emitted by the lamp is within the normal range. Multiple pumps can be individually controlled to achieve different flow rates, which can be detected by flow meters. Treated milk can be collected after each coil of treatment at the aseptic sampling points 1 and 2. In this study, 80%, 300% and 500% UVC-treated milk was taken at point 1, and 1000% UVC-treated milk was collected at point 2.

The UVC treatment was conducted using a double-pass UVC system designed and produced by Lyras (Aalborg, Denmark), with a process design as shown in Fig. 2. Raw milk was fed into tank 1 and pumped into the plastic coil (food-grade fluorinated ethylene propylene (FEP), internal diameter: 7 mm). The flow rate (Q) was set to 400L/h, which resulted in a retention time of 5.8 s for the milk to pass through each coil. The milk flow was irradiated perpendicularly by UV light emitted from the low-pressure mercury lamp (220W, 38% of output at 254 nm wavelength). A patented UVC bandpass filter coated onto quartz glass was installed between the lamp and coil to filter away light at wavelengths higher than 254 nm, primarily in the UV-B area. This system can operate in both fully turbulent and transitional/dean vortex regimes to ensure a proper mixing of milk, which in turn achieves a homogenous exposure to UVC light. The Reynolds number of this system. The Reynolds number of the milk in this system was calculated using equation (1).

(1) $$Re=\frac{\rho \times v\times D}{\mu }$$

Where ρ is the milk density (1030 kg/m^3^), v is the linear flow velocity, D is the diameter of the coil (7 mm), and μ is the dynamic viscosity (0.002 Pa s). The linear flow velocity was calculated from the volumetric flow rate (400L/h) using (v=Q/A), where ($A=\pi D^{2}/4$) is the cross-sectional area of the tube. Therefore, the Reynolds number is 1.04 × 10^4^. Since it's greater than 4000, the flow regime is turbulent (Bertsche et al., 2016). After the milk was pumped through the second coil, it was collected into tank 2. For higher UVC-dose-treated samples, the milk in tank 2 was transported back to the balance tank for multiple cycles of treatment. The treatment performed under maximum light intensity is referred to as the 100% treatment. In industrial practice, milk is typically treated at 80% light intensity, as this level achieves a comparable microbial reduction effect to that of high-temperature short-time (HTST) pasteurization based on previous trials. Four UVC-treated milks were created: one was treated once with 80% exposure, and the other three were treated 3, 5, and 10 times with 100% exposure, respectively. These samples were named UV80, UV300, UV500, and UV1000. The incidence of fluence (H_0_, kJ/L) was calculated with equation (2).

(2) $$H_{0}\;\left(J/L\right)=H\times C\times N$$

Where H is the volumetric UV dose (0.48 kJ/L) measured by Fiege et al. (2022), C is the light intensity (%), t is the retention time through the coil (5.8 s), and N is the pass number. Therefore, the incidence of fluence of samples UV80, UV300, UV500 and UV1000 were 0.384, 1.44, 2.4 and 4.8 kJ/L, respectively. UV0 is the untreated, raw milk sample.

During UVC treatment, the outlet temperature was not continuously recorded. A conservative theoretical calculation was performed based on the applied 254 nm volumetric doses. Assuming that 38% of the emitted lamp energy contributed to the 254 nm UVC dose and that all emitted energy was converted into heat in the liquid, the temperature increase was estimated as ΔT = dose at 254 nm/(0.38 × 4186), where 4186 J/kg·°C is the specific heat capacity of water. Based on this calculation, the estimated temperature increases were approximately 0.24, 0.91, 1.51, and 3.02 °C for UV80, UV300, UV500, and UV1000, respectively. These values indicate that direct heating due to UV irradiation was limited under the applied conditions.

#### 2.2.2 Nano-second pulsed electric field treatment

**Fig. 3:**
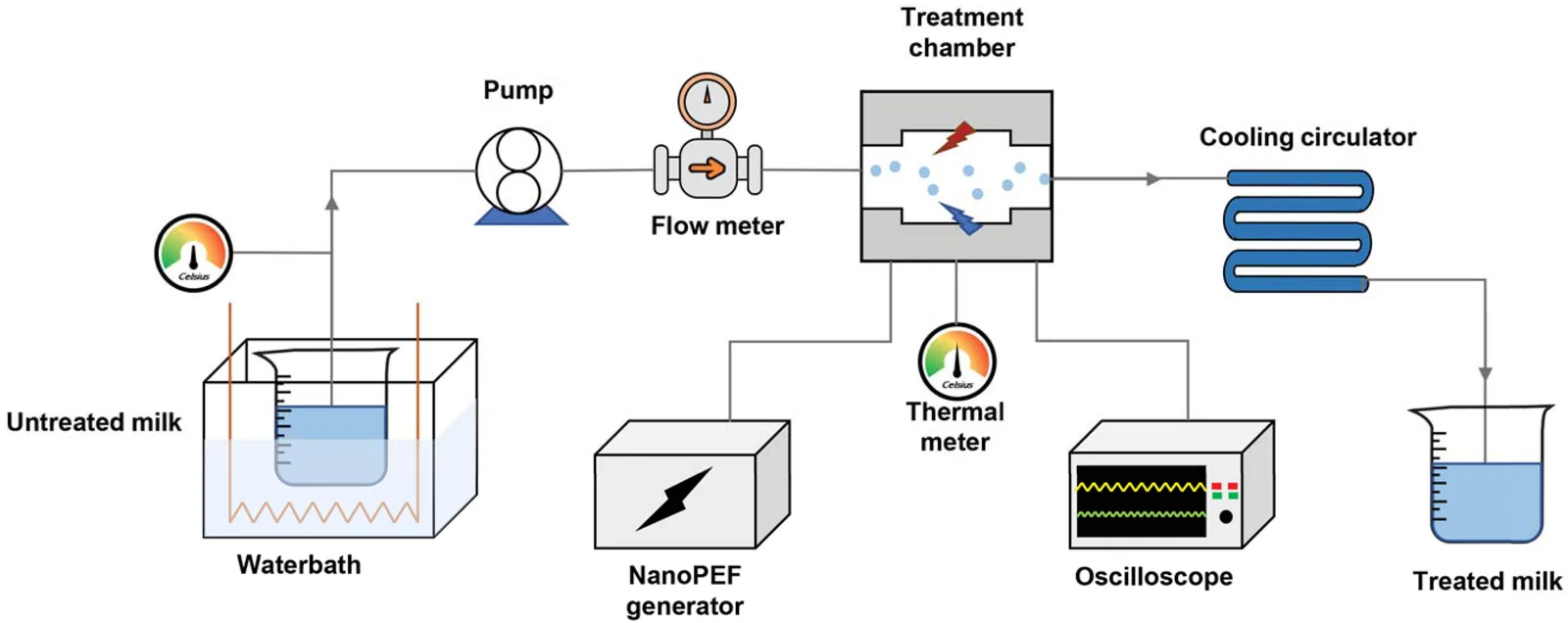
A schematic view of the nanoPEF system. A waterbath is used to gently preheat the milk. The flow rate of pumped milk is measured by a flow meter. The actual applied voltage across the treatment chamber is measured by a high-voltage probe connected to an oscilloscope. Inlet and outlet temperatures are controlled and measured with inline temperature-measuring sensors integrated into the processing circuit.

As shown in Fig. 3, milk was PEF-treated in a continuous flow chamber using the equipment designed by PEF Technologies B.V. (Enschede, The Netherlands). The milk was pumped through the treatment chamber with an arrangement of plane parallel electrodes with a gap of 0.3 cm width. Exponential decay monopolar pulses of 150-160 ns width were delivered at electric field strengths of 60-65 kV/cm and a frequency of 60 Hz to reach a total treatment time of about 1.3 μs. The energy of density (E, kJ/L) was calculated with equation (3).

(3) $$E\;\left(kJ/L\right)=\frac{Epp\times Np\times F\times 3600}{Q}$$

Where Epp is energy per pulse (8.05 J), F is frequency (Hz), and Q is flow rate (L/h). Therefore, under the settings used, the milk was treated by an energy density of 29 kJ/L. To obtain the samples with higher energy input, both higher initial temperature (T_in_, °C) and pulse numbers (Np) were applied, as shown in Table 1. These treatments corresponded to a total specific energy ranging from 20 to 190 kJ/L of nanoPEF-treated milk. The milk was immediately cooled to 10 °C with a cooling circulator after treatments. P0 is the reference original raw milk sample, without treatment or pre-heating.

**Table 1:** NanoPEF treatment conditions.

Sample	F, Hz	ES, kV/cm	Q, L/h	Np	E, kJ/L	T_in_, °C	T_out_, °C
P1	60	60-65	60	8	29	43	50
P2	60	60-65	60	8	29	51	58
P3	60	60-65	60	8	29	60	65
P4	60	60-65	60	58	182	20	65

ES is the field strength across the treatment chamber; T_in_ and T_out_ represent the temperatures of the sample entering and exiting the treatment chamber, respectively.

### 2.3 Total aerobic mesophilic and thermoduric microorganism counts and coliform counts

To assess the microbial levels in raw and treated milk samples, total aerobic mesophilic and thermoduric microorganism counts were determined according to the methods of NEN-EN-ISO 4833-1 and NEN 6807, respectively. The two methods differ slightly in that for the thermoduric microorganism's measurement, milk was pasteurized at 63.5 °C for 30 min before spreading on plates. In brief, 1 mL of the milk or a decimal dilution was spread on the plate prepared using Plate Count Milk Agar. The plates were aerobically incubated at 30 °C for 72 h. Coliform counts were measured as reported previously (Makararpong et al., 2024). Then, a serially diluted milk sample was plated by spreading 1 mL onto Violet Red Bile Agar plates and incubated at 35 °C for 24 h. After the incubation, the number of microorganisms per mL was calculated from the number of colonies obtained on selected plates (10-300 colonies per plate). Based on the plating of 1 mL of the lowest dilution, the limit of detection (LOD) was 1 CFU/mL, and the limit of quantification (LOQ) was 10 CFU/mL. Samples with no colonies observed on the lowest dilution plate were reported as not detected (ND).

### 2.4 Skim milk and milk serum preparation

After treatments, skim milk and milk serum were prepared. To remove the fat, milk samples were centrifuged at 6414 x g for 30 min at 4 °C (Sorvall LYNX 6000, Thermo Fisher Scientific, USA). Then, the pH of part of the skim milk was adjusted to 4.6 with 1 M hydrochloric acid (HCl) to precipitate the caseins and denatured milk serum proteins (Law and Leaver, 2000). Subsequently, the acidified skim milk was transferred to ultracentrifuge tubes and left for 30 min to equilibrate. After that, they were ultracentrifuged at 100,300 x g for 90 min at 30 °C. The skim milk and milk serum were stored at −20 °C for further analysis.

### 2.5 Native protein content

In this study, native milk serum proteins are defined as those proteins remaining in the milk serum fraction after pH 4.6 precipitation and ultracentrifugation. This method is based on Law and Leaver (2000), who showed that denatured and aggregated whey proteins can be removed under these conditions. The native protein content of the milk serum fraction was determined by a DUMAS Flash EA 1112 Protein analyser (Thermo Fisher Scientific, USA). Nitrogen content was converted into protein content by applying a nitrogen-to-protein conversion factor of 6.38. The protein concentrations were converted to mg/mL of milk serum.

### 2.6 SDS-PAGE of the milk serum fraction

To determine the milk serum protein composition and aggregation level, SDS-PAGE under both reducing and non-reducing conditions was performed. For the reducing conditions, 2 μL sample, 5 μL sample buffer, 2 μL reducing agent and 13 μL water were mixed and centrifuged at 2000 rpm for 1 min. For the non-reducing conditions, the reducing agent was replaced by MilliQ water. Samples were heated at 70 °C for 10 min and centrifuged again at 2000 rpm for 1 min. Then, 4 μL of marker and 10 μL of samples were loaded on 12% Bis-Tris gels and run in the MOPS running buffer for around 75 min. After running, gels were stained with Coomassie Brilliant Blue, followed by washing the gel with 10% ethanol and 7.5% acetic acid in MilliQ water to remove the background colour. The gels were scanned with a gel scanner (Biorad - GS900, USA). The analysis of the scanned gels was performed with Image Lab software (v6.1) by comparing the relative intensities of the bands.

### 2.7 Identification and quantification of milk serum proteins by LC-MS/MS

#### 2.7.1 Filter-aided sample preparation (FASP) for LC-MS/MS

Filter-aided sample preparation was performed as reported (Y. Liu et al., 2020; Wiśniewski et al., 2009) with some modifications. In brief, the milk serum samples were diluted 8 times in 100 mM Tris pH 8 to a protein concentration of around 1 μg/μL. Then, 80 μL of these samples were mixed with 8 μL 150 mM dithiothreitol and incubated at 45 °C for 30 min in a thermomixer to achieve protein reduction. Next, 176 μL freshly prepared 100 mM Tris pH 8 in 8M urea and 26.4 μL 200 mM acrylamide were added and incubated for 10 min at room temperature while mildly shaking to unfold and alkylate proteins. Thereafter, 145 μL alkylated sample was transferred to an ethanol-washed Pall 3K omega filter (3 kDa cut-off, OD003C34, Pall Corporation, USA) and centrifuged at 12,000 rpm for 45 min. The filter was washed twice with 150 μL 2M Ammonium bicarbonate (NH_4_HCO_3_) and 100 μL 70% ethanol, respectively. To digest proteins on the filter, 100 μL 5 ng/μL trypsin (diluted with 50 mM NH_4_HCO_3_) was added to the filter, followed by overnight incubation while mildly shaking at room temperature. Then, the peptides were centrifuged through the filter at 12,000 rpm for 30 min, followed by the addition of 100 μL 1 mL/L formic acid (HCOOH) in water onto the filter and another 30 min centrifugation. Finally, 10% trifluoroacetic acid was added to the filtrate to adjust the pH to around 3. The samples were stored at −20 °C before being measured by LC-MS/MS.

#### 2.7.2 LC-MS/MS analysis

The LC-MS/MS analysis of the peptide samples was conducted as described previously (Kontopodi et al., 2022). In brief, the peptide samples were injected onto a 0.10 × 250 mm ReproSil Saphir 100 C18 1.5 μm beads analytical column (made in-house) at a pressure of 800 bar. Peptides were eluted using a gradient elution method with acetonitrile, ranging from 9% to 34% in water containing 0.1 mL/L formic acid, at a flow rate of 0.5 μL/min for 50 min. Then, an electrospray potential of 3.5 kV was applied to the eluent through a stainless-steel needle fitted into the P777 Upchurch micro cross waste line, positioned between the pump and the analytical column. The Orbitrap Exploris 480 mass spectrometer (Thermo Fisher Scientific, Waltham, MA, USA) was used to measure full MS and MS/MS scans in (data-dependent) positive mode between 380 and 1400 m/z. The most intense precursor ions from the MS1 scan were isolated and fragmented. Higher-energy collisional dissociation (HCD) was applied to fragment precursor ions to record the peaks of these ions in data-dependent acquisition (DDA) mode. The Uniprot *Bos taurus* protein database (UP000009136), together with a contaminants database, was used. Proteins were identified and quantified by MaxQuant (2.0.3.0). The FDR cutoff was set to 0.01. The minimum peptide length was set to seven, and the maximum number of missed cleavages was set to two. Propionamide (C) (fixed), oxidation (M) (variable), and deamidation (NQ) (variable) were set for searching protein modifications. Potential contaminants, except for those from bovine origin, and proteins detected without a unique peptide, were removed from the results.

### 2.8 Surface hydrophobicity measurement

The surface hydrophobicity was measured according to a published method (Lam and Nickerson, 2015) with adjustments. Briefly, milk serum was diluted to 0.2, 0.4, 0.6, and 0.8 mg/mL with water. The mixture of 2 mL of diluted milk serum and 100 μL of 8 mM ANS was incubated in the dark at 37 °C for 15 min. Then, the fluorescence was measured by applying excitation and emission wavelengths at 390 nm and 490 nm, respectively. The gradient of fluorescence intensity against protein concentration was used as the surface hydrophobicity.

### 2.9 Accessible and total thiol group content

Accessible and total thiol group content were measured following the method of Fitzner et al. (2023). The accessible thiol groups were measured by mixing 800 μL of treated skim milk, 800 μL of Tris-glycine buffer (50 mM, pH 8.5), and 40 μL of 10 mM DTNB in Tris-glycine buffer. For the total thiol groups measurement, 8M urea was added to the Tris-glycine buffer to denature the proteins and expose the buried thiols. Then, the samples were incubated for 10 min. The absorbance was measured at 412 nm. Calibration curves of L-cysteine with and without urea were made to calculate the thiol group content.

### 2.10 Free amino group content

To determine the potential protein fragmentation, free amino group content was determined using the OPA assay as described previously (L. Liu et al., 2022). OPA reagent was freshly prepared in a total volume of 100 mL by mixing 3.81g sodium tetraborate decahydrate, 0.088g DTT, 0.1g SDS, and 0.080g OPA dissolved in 4 mL ethanol. In addition, an L-serine standard curve ranging from 5 to 50 μM was prepared. Then, 200 μl freshly prepared OPA reagent was mixed with either 10 μl milk serum sample, acid hydrolysate or L-leucine standard and incubated in the dark at room temperature for 15 min. The absorbance at 340 nm was measured by a microplate reader (SpectraMax ABS Plus, Molecular Devices, USA). The free amino group content was calculated in mmol per g of protein.

### 2.11 Protein-bound carbonyl content

Protein-bound carbonyl content was determined as an indicator of the oxidation state of the proteins, using the method adapted from published studies (Fitzner et al., 2023; Scheidegger et al., 2010). First, 400 μL of the milk serum fraction was mixed with 1.5 mL 10 mM 2,4-dinitrophenylhydrazine (DNPH) in 2M HCl and incubated in the dark at room temperature for 45 min. Proteins in the samples were then precipitated with 2 mL 10% (w/v) trichloroacetic acid (TCA) and left for 30 min, after which samples were centrifuged at 10,000 rpm for 5 min. The obtained protein pellets were washed with 1 mL 1:1 ethanol/ethyl acetate (v/v) and centrifuged again at 10,000 rpm for 10 min. The washing was repeated three times to remove the residual DNPH. Proteins were redissolved in 1 mL of 6M guanidine-HCl (pH 2.3). Absorbance at 370 nm was recorded by the spectrophotometer. Carbonyl content was determined using the molar extinction coefficient (*ε*) 21,000 M^−1^ cm^−1^ (Feng et al., 2015). The carbonyl content was calculated into μmol per g of protein based on equation (4).

(4) $$Carbonyl\;\left(\mu mol/g\right)=\frac{A370}{\varepsilon \times L}$$

Where A370 is the UV absorbance at 370 nm, *ε* is the molar extinction coefficient (21,000 M^−1^ cm^−1^), and L is the width of the cuvettes (10 mm).

### 2.12 Fluorescent oxidation product: N-formylkynurenine (NFK) and dityrosine contents

NFK and dityrosine contents were determined as indicators for the oxidation state of the proteins, using a procedure adapted from published studies (Fitzner et al., 2023; Scheidegger et al., 2016). Milk serum samples were diluted to 7 mg/mL using 6M pH 7 guanidine hydrochloride solution, and the samples were left at room temperature for 1 h to complete denaturation. Measurements were done using a fluorophotometer (Shimadzu, Japan) with a 5 nm bandwidth and 10 ms accumulation time. The formation of dityrosine was monitored by the increase in fluorescence using excitation and emission wavelengths of 325 and 410 nm. The formation of N-formylkynurenine was monitored by excitation and emission wavelengths of 325 and 435 nm, respectively. The readings were given in arbitrary units (arb.u.) per mg of protein.

### 2.13 Statistical analysis

For proteomics, the intensity-based absolute quantification (iBAQ) was determined by MaxQuant (Boland and Hill, 2019). The iBAQ value reflects the summed intensities of all peptides identified for a given protein, normalized by the number of theoretically observable peptides. After log_(10)_ transformation of the iBAQ values, principal component analysis (PCA) was performed using the R package ‘prcomp’ and plotted using the package ‘ggbiplot’. The bubble plot of protein concentrations and numbers was created using the R package ‘ggplot’. The Hierarchical clustering of immune-related proteins was created with the R package ‘pheatmap’. The volcano plot between P0 and P4 samples was analyzed using the ‘limma’ package in R. Protein intensities were log2-transformed prior to statistical analysis. A linear model was fitted for each protein, and empirical Bayes moderation was applied to stabilize variance estimates across proteins. P-values were adjusted for multiple testing using the Benjamini–Hochberg false discovery rate (FDR) method. Proteins with an absolute log2 fold change (|log2FC|) ≥ 1 and an adjusted p-value (FDR) < 0.05 were considered significantly differentially changed. The immune-related proteins mentioned in the bubble plots were classified by gene ontology (GO) Biological Process (BP) using the online tool Panther (https://www.pantherdb.org/). For the data analysis of the other measurements, one-way ANOVA (SPSS 30.0.0) and Turkey's HSD post-hoc testing were performed for multiple means comparisons and determination of significant differences among the different treatments. For all results, *p* < 0.05 was considered statistically significant. Three independent biological replicates and three technical replicates were conducted. Data was presented as mean ± standard deviation of triplicates.

## 3 Results and discussion

In this study, UVC and nanoPEF were evaluated as two non-thermal processing strategies with distinct mechanisms. Therefore, the results are presented primarily within each treatment relative to its corresponding untreated control as well as compared to the high-intensity pasteurized sample.

### 3.1 Reduction of microorganisms

The aerobic mesophilic and thermoduric microorganisms and coliform counts are shown in Table 2. High-intensity pasteurization (85 °C 5 min, abbreviated as 85_5 in the following text) achieved a significant 3.0-log and 1.8-log reduction in naturally occurring aerobic mesophilic and thermoduric microorganisms, respectively (*p* < 0.05). Although both UV 80 and nanoPEF P1 treatments significantly decreased the total bacterial count in raw milk (*p* < 0.05), their effectiveness remained lower than that of the 85_5 heat treatment. The log reduction of aerobic mesophilic bacteria was within or above the range reported for high-temperature-short time pasteurization (HTST, 2.3-log reduction) in a previous study (Z. Li et al., 2021). UV300, P2 and P3 treatments resulted in a ∼3-log and ∼1.8-log reduction in aerobic mesophilic and thermoduric microorganisms, comparable to that of the 85_5 treatment (*p* ≥ 0.05). With higher treatment intensity, UV500, UV1000, and P4 resulted in greater reduction than 85_5 (p < 0.05), especially for thermoduric microorganisms. This indicates that the reduction of naturally occurring microbes by UVC and nanoPEF increased with more intense treatment conditions. Heat treatments generally inactivate microorganisms through thermal damage to cellular proteins, membranes, and other essential macromolecules under sustained heat stress (Smelt and Brul, 2014). However, thermoduric bacteria or stress-resistant cells may survive heat treatment, which can contribute to bacterial counts after processing (Owusu-Darko et al., 2019; Ranieri et al., 2009). In the present study, UVC and nanoPEF treatments were associated with only limited temperature increases, suggesting that their microbial reductions were not primarily driven by bulk heating. For UVC, microbial inactivation is generally attributed to photochemical damage to nucleic acids, particularly the formation of DNA photoproducts that inhibit replication. Additional oxidative damage to cellular components may also occur, although such mechanisms were not directly assessed in the present study (Petersen, 2017). For nanoPEF, microbial inactivation has been linked to membrane permeabilization, electroporation-related damage, and possible intracellular effects induced by ultrashort high-intensity electric field pulses. A higher initial temperature (still below 55 °C) increases the electrical conductivity of milk, thereby lowering its resistance, raising the current, reducing the critical electric field intensity required for microbial Inactivation, and promoting molecular transport (Araújo et al., 2023; Yan et al., 2021). Therefore, the microbial reductions observed in this study are consistent with the known inactivation principles of UVC and nanoPEF, although the specific cellular damage mechanisms were not directly determined. In addition, the reduction of coliforms in the raw milk was successful with high-intensity pasteurization, UVC and nanoPEF treatments. Even under the least intense UVC and nanoPEF treatment conditions, the coliform count was reduced to an undetectable level.

**Table 2:** Total aerobic mesophilic, thermoduric bacteria, and coliform counts and corresponding log reduction in whole milk before and after treatments of UVC, nanoPEF and high-intensity pasteurization.

Treatments	Conditions	Microorganism counts (Log10 CFU/mL)		
		Aerobic mesophilic bacteria	Aerobic thermoduric bacteria	Coliform
UVC	0	5.94 ± 0.12 ^a^	3.39 ± 0.04 ^a^	2.02 ± 0.12
	80%	3.17 ± 0.38 ^b^ (2.77)	1,95 ± 0,06 ^b^ (1.44)	ND
	300%	2.68 ± 0.06 ^c^ (3.26)	1.56 ± 0.05 ^bc^ (1.83)	ND
	500%	2.06 ± 0.08 ^cd^ (3.88)	1.25 ± 0.07 ^c^ (2.14)	ND
	1000%	1.58 ± 0.27 ^d^ (4.36)	0.97 ± 0.85 ^c^ (2.42)	ND
nanoPEF	P0	5.76 ± 0.11 ^A^	3.45 ± 0.14 ^A^	1.85 ± 0.14
	P1	3.49 ± 0.12 ^B^ (2.27)	1.92 ± 0.05 ^B^ (1.53)	ND
	P2	2.86 ± 0.06 ^C^ (2.90)	1.63 ± 0.10 ^B^ (1.82)	ND
	P3	2.39 ± 0.14 ^D^ (3.37)	1.46 ± 0.10 ^BC^ (1.99)	ND
	P4	1.80 ± 0.12 ^E^ (3.96)	0.86 ± 0.18 ^C^ (2.59)	ND
High-intensity pasteurization	85 °C, 5 min	2.90 ± 0.16 ^bc^ (3.04)	1.57 ± 0.16 ^b^ (1.82)	ND

Values in parentheses indicate log reductions compared with the corresponding untreated control within each treatment series: U0 for UVC-treated samples and P0 for nanoPEF-treated samples. Different lowercase letters (a–e) indicate significant differences among the UVC-treated samples and the 85_5 sample, whereas different uppercase letters (A–D) indicate significant differences among the nanoPEF-treated samples. Comparisons between aerobic mesophilic bacteria and aerobic thermoduric bacteria were conducted separately. ND = non-detected; no colonies were observed on the lowest dilution plate.

Thus, UVC and nanoPEF showed treatment-intensity-dependent reductions in naturally occurring microbes. Reductions in aerobic mesophilic counts were within the range reported for HTST-treated milk when UVC and nanoPEF intensities were above approximately 0.48 kJ/L (UV80) and 29 kJ/L (P1), respectively. To achieve inactivation similar to high-intensity pasteurization, UVC and nanoPEF treatments at approximately 1.44 kJ/L (UV300) and 29 kJ/L (P2), respectively, were needed. The interpretation of the microbial results is limited by the scope of the naturally occurring microbial analyses. Targeted challenge tests or similar tests should be performed before pasteurization-level safety can be claimed.

### 3.2 Native milk serum protein content and SDS-PAGE profile

**Fig. 4:**
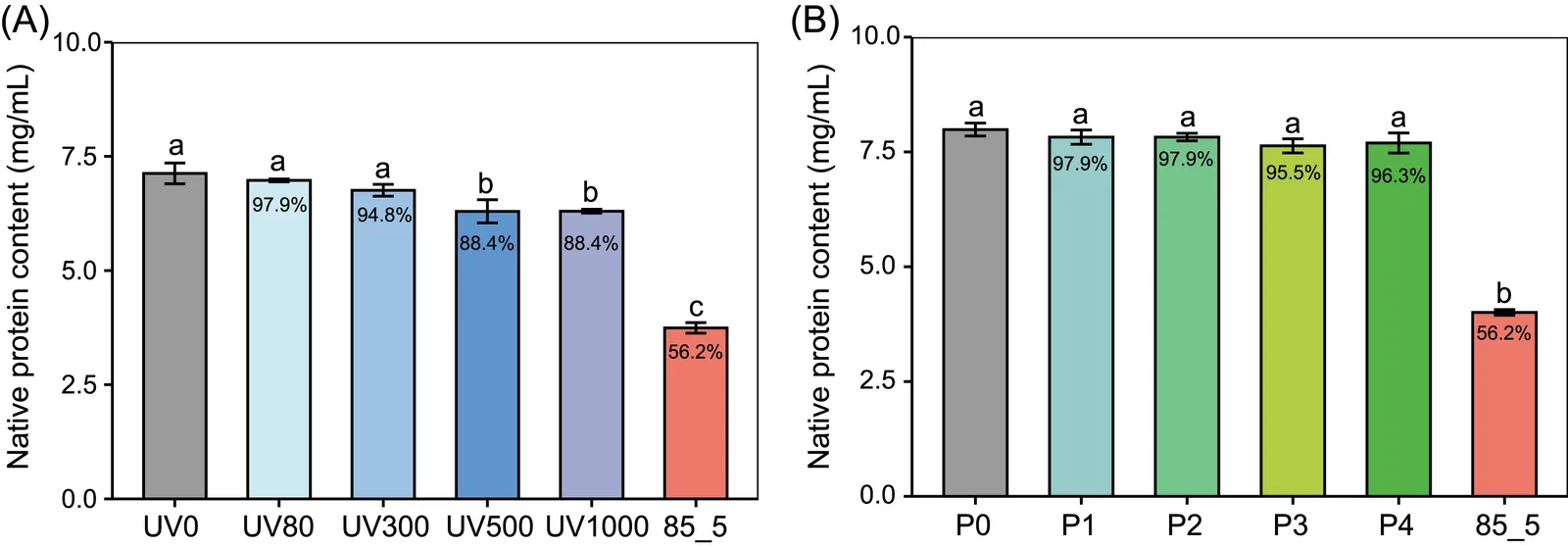
Native milk serum protein content of UVC and 85_5 (A); nanoPEF and 85_5 (B) measured by the DUMAS method. UV0-UV1000: UV0 is raw milk, UV80-UV1000 are milk irradiated from low to high intensity UVC; P0-P4: P0 is raw milk, P2-P4 are milk treated with low to high intensity nanoPEF; 85_5 is heated at 85 °C for 5 min. The numbers on the bars show the relative protein content compared with UV0 and P0, respectively. a-c means significant differences (*p* < 0.05). Data are presented as mean ± SD (n = 3 independent processing replicates).

**Fig. 5:**
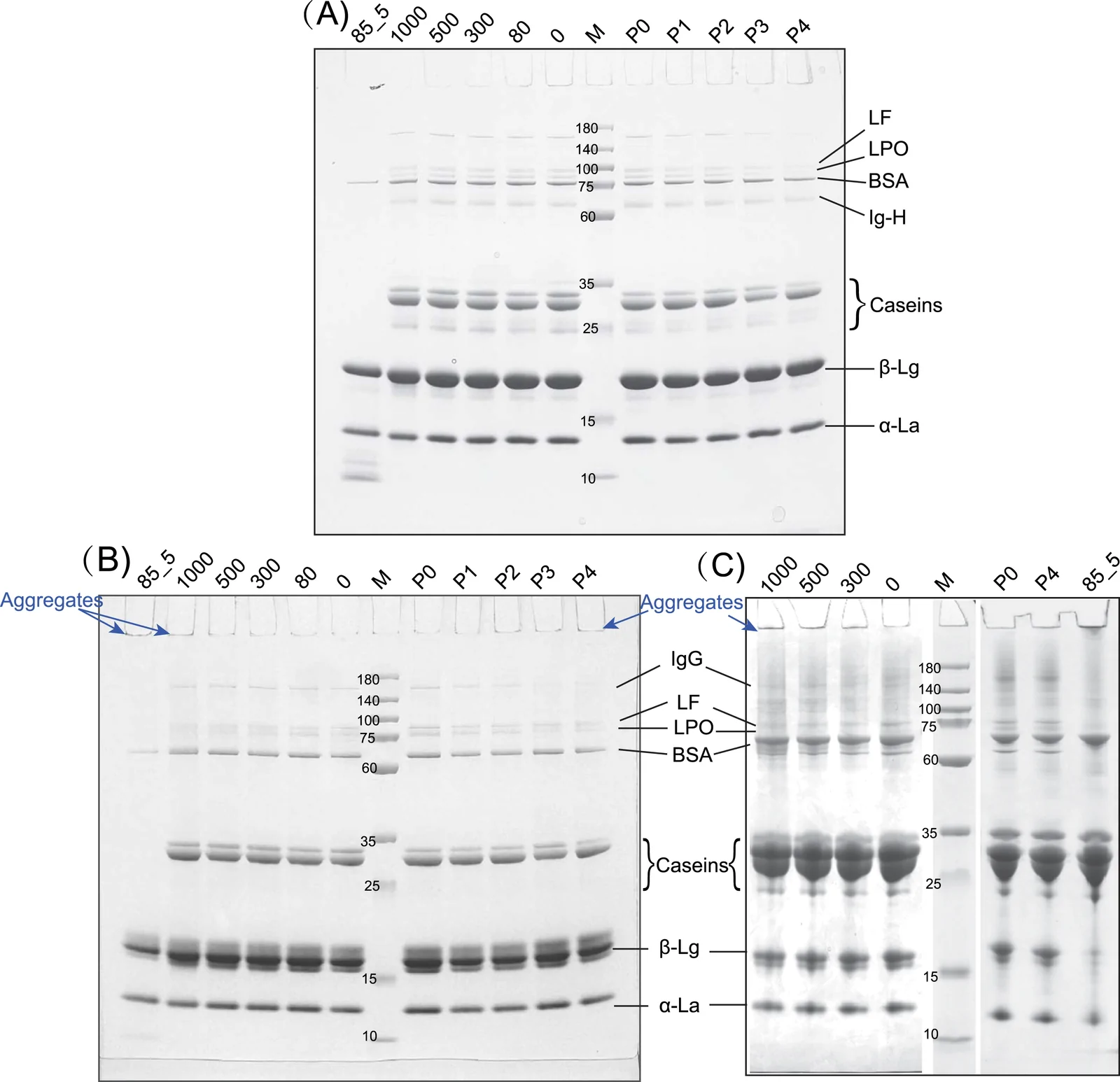
SDS-PAGE of milk serum proteins under reducing (A) and non-reducing (B) conditions, and of skim milk samples under non-reducing conditions (C). M: protein marker; 0-1000: UV0-UV1000: UV0 is raw milk, UV80-UV1000 are milk irradiated from low to high intensity UVC; P0-P4: P0 is raw milk, P2-P4 are milk treated with low to high intensity nanoPEF; 85_5 is heated at 85 °C for 5 min. IgG: immunoglobulin G; LF: lactoferrin; LPO: lactoperoxidase; BSA: bovine serum albumin; Ig-H: IgG-heavy chain; β-Lg: β-lactoglobulin; α-La: α-lactalbumin. Numbers on the bands of the marker indicate molecular weights.

The total milk serum protein concentration was measured using the DUMAS method, with the results shown in Fig. 4. In the 85_5 sample, the milk serum protein content significantly decreased from 7.1 mg/mL to 4.01 mg/mL (*p* < 0.05), meaning that approximately 56% of the native protein was retained in the serum. This result is consistent with the findings of Brick et al. (2017), who reported that heating milk serum protein at 80 °C for 5 min or at 100 °C for 30 s resulted in a 51% reduction in its content. This reduction occurs because milk serum proteins gradually denature when exposed to temperatures above 65 °C, with more intense heating leading to subsequent aggregation (Abbring et al., 2020). At pH 4.6, caseins as well as denatured and aggregated whey proteins precipitate, while native serum proteins remain soluble (Law and Leaver, 2000). Denatured/aggregated milk serum proteins may associate with caseins due to disulfide bridging, hydrophobic interactions, and the absence of electrostatic repulsion (de Wit and Kessel, 1996). Therefore, they are expected to be largely removed together with caseins. Compared to heated samples, UVC and nanoPEF were more effective in preserving native milk serum proteins. In samples treated by low-dose UVC (80% and 300%) and all nanoPEF conditions, the native milk serum protein content showed no significant difference from that of untreated milk. However, in the UV500 and UV1000 samples, the native milk serum protein content significantly decreased by ∼12% (*p* < 0.05), suggesting that these intense UVC treatments may have caused limited protein denaturation. In the study conducted by Kontopodi et al. (2022), no significant decrease in native milk serum protein content was observed after UVC irradiation, as limited UVC dosages (2.43-4.86 kJ/L) were used. At comparable UVC intensities, the UVC filter used in this system ensured a higher efficiency of UVC irradiation. This further confirms that protein denaturation increases with higher UVC dosages. To get a first indication about the effect of these treatments on the most abundant milk serum proteins, including their retention and aggregation, reducing and non-reducing SDS-PAGE were performed on both the milk serum fraction and skim milk (Fig. 5).

Under non-reducing conditions (Fig. 5B), IgG existed in the native quaternary structure because the disulfide bonds were not cleaved (H. Liu & May, 2012)**.** Under reducing conditions, the abundant milk serum protein bands remained visible in all UVC and nanoPEF-treated samples, while the intensity of these bands slightly decreased as the treatment intensity increased. In contrast, only small amounts of the relatively highly abundant milk serum proteins, BSA, α-La, and β-Lg, were present in the heat-treated samples, which supports the DUMAS results (Fig. 4). A similar trend was observed on the gel under non-reducing conditions. In the UV1000 and P4 samples, all protein bands became fainter, with faint protein smears across the gel lane. Additionally, an aggregate band appeared in the wells of these samples as well as in the heated sample. These changes observed in the most intensely treated samples may reflect the formation of protein aggregates, potentially involving disulfide-mediated cross-linking, with part of these aggregates being removed during acidification and ultracentrifugation. To further investigate the aggregation behaviour, SDS-PAGE of skim milk under non-reducing conditions was conducted (Fig. 5C). Compared to raw milk, the milk serum protein bands’ intensities in the 85_5 sample decreased sharply, particularly for α-lactalbumin and β-lactoglobulin, with a greater accumulation of aggregates at the top of the gel. Notably, the casein content remained nearly unchanged in all skim milk samples (Fig. 5C); however, no casein was detected in the heated milk serum (Fig. 5A & B). One possible explanation for these observations is the involvement of thiol–disulfide exchange reactions, as κ-casein and multiple thiol groups located on cysteine residues in milk serum proteins, particularly β-lactoglobulin (β-Lg) (Morand et al., 2011). These thiol groups are exposed during protein unfolding and/or rearrangement. They potentially trigger intra- and intermolecular disulfide bond formation and reshuffling, promoting protein aggregation between milk serum proteins and caseins (Lowe et al., 2004; Manzo et al., 2015). Heating promoted this aggregation, thus more caseins and whey proteins were removed after precipitation. In contrast, this phenomenon was barely observed in P1–P3, as the increase in treatment intensity was achieved solely by raising the temperature to below 65 °C, which was insufficient to induce disulfide bond rearrangement. When a higher electric field energy and initial temperature were applied to P4, a small amount of protein aggregation occurred. Similarly, aggregation was only observed under higher UVC irradiation doses (500%–1000%). These observations suggest that disulfide-mediated aggregation may become more apparent at the highest UVC and nanoPEF treatment intensities.

Altogether, the above results indicate that UVC and nanoPEF better preserved native milk serum proteins compared to heat treatment. The most intense UVC dosage and nanoPEF energy impacted disulfide bond reconstitution, leading to minor protein aggregation. However, these changes were considerably smaller than those observed in the heated sample. To further validate and explain this, structural changes in the proteins were also investigated, as will be described in the next section.

### 3.3 Milk serum protein structural changes: unfolding and aggregation

#### 3.3.1 Surface hydrophobicity (S_0_)

**Fig. 6:**
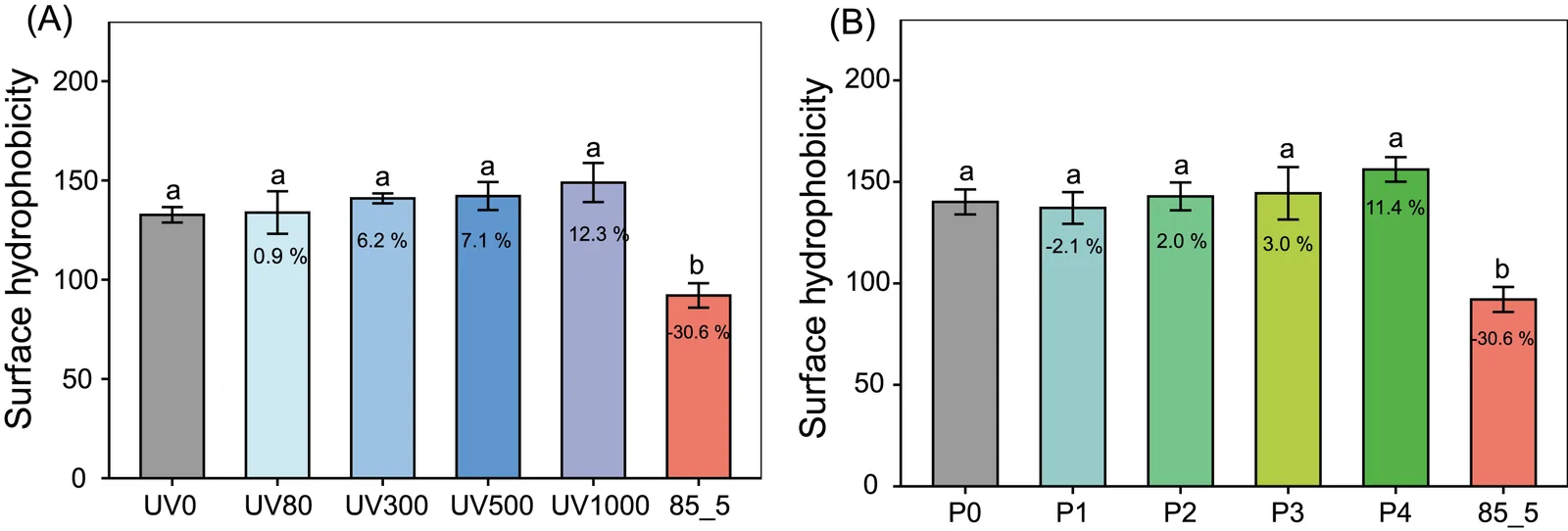
Surface hydrophobicity (S_0_) of milk serum after UVC, 85_5 (A), and nanoPEF (B) treatments. a-b means significant differences (*p* < 0.05). The numbers on the bars show how much the surface hydrophobicity of the treated samples increased compared with UV0 and P0, respectively. The 85_5 sample was made from the UV0 sample; therefore, its hydrophobicity value was compared with that of UV0. UV0-UV1000: UV0 is raw milk, UV80-UV1000 are milk irradiated from low to high intensity UVC; P0-P4: P0 is raw milk, P2-P4 are milk treated with low to high intensity nanoPEF; 85_5 is heated at 85 °C for 5 min. Data are presented as mean ± SD (n = 3 independent processing replicates).

The unfolding of globular proteins during denaturation exposes hydrophobic residues (e.g., tryptophan, glycine, phenylalanine), making surface hydrophobicity (S_0_) a key indicator of milk serum protein unfolding and denaturation extent. As illustrated in Fig. 6, UVC and nanoPEF did not induce significant changes in surface hydrophobicity (*p* > 0.05). Even under the most intense treatment conditions (UV1000 and P4), hydrophobicity increased only slightly by 11.2% and 12.4%, respectively. This suggests that only a very limited extent of milk serum protein unfolding occurred, with most milk serum proteins maintaining their native structure. This finding is consistent with the results of native milk serum protein content (Fig. 4). Similarly, Sui et al. (2011) reported a 11.4% increase in surface hydrophobicity when applying microsecond PEF to a 10% (w/w) whey protein isolate sample at a lower electric field intensity (131.9 kJ/L). Notably, compared to their study, an electric field intensity 1.4-fold as high (182 kJ/L) was used in this study to enhance microbial inactivation efficiency, yet no significantly higher surface hydrophobicity was observed. This is likely due to the shorter processing time and higher electric pulse efficiency of nanoPEF. Additionally, similar UV study (Fitzner et al., 2023) has reported that UVB irradiation at an equivalent dosage (3 J/cm^2^) led to a 9% increase in β-lactoglobulin hydrophobicity, which aligns with our findings.

In contrast, the surface hydrophobicity of milk serum decreased significantly by 30.6% (*p* < 0.05) after heating at 85 °C for 5 min compared to the untreated sample. This reduction is attributed to the intense heat-induced unfolding of milk serum proteins, leading to the excessive exposure of hydrophobic sites, which in turn promotes intermolecular covalent bonding and aggregation. This is confirmed by SDS-PAGE (Fig. 5). Although larger aggregates were removed by ultracentrifugation, soluble aggregates remained in the milk serum. This observation is consistent with a previous study (Yüksel and Erdem, 2005). They reported that the number of surface hydrophobic sites in β-lactoglobulin increased at 80 °C for 5 min, attributed to a more relaxed protein structure. However, it began to decrease at 90 °C for 5 min due to structural rearrangement and aggregation. These observations confirm that although UVC and nanoPEF have a greater power for cell damage than heating, their impact on protein structure is minimal.

#### 3.3.2 Thiol group content

**Fig. 7:**
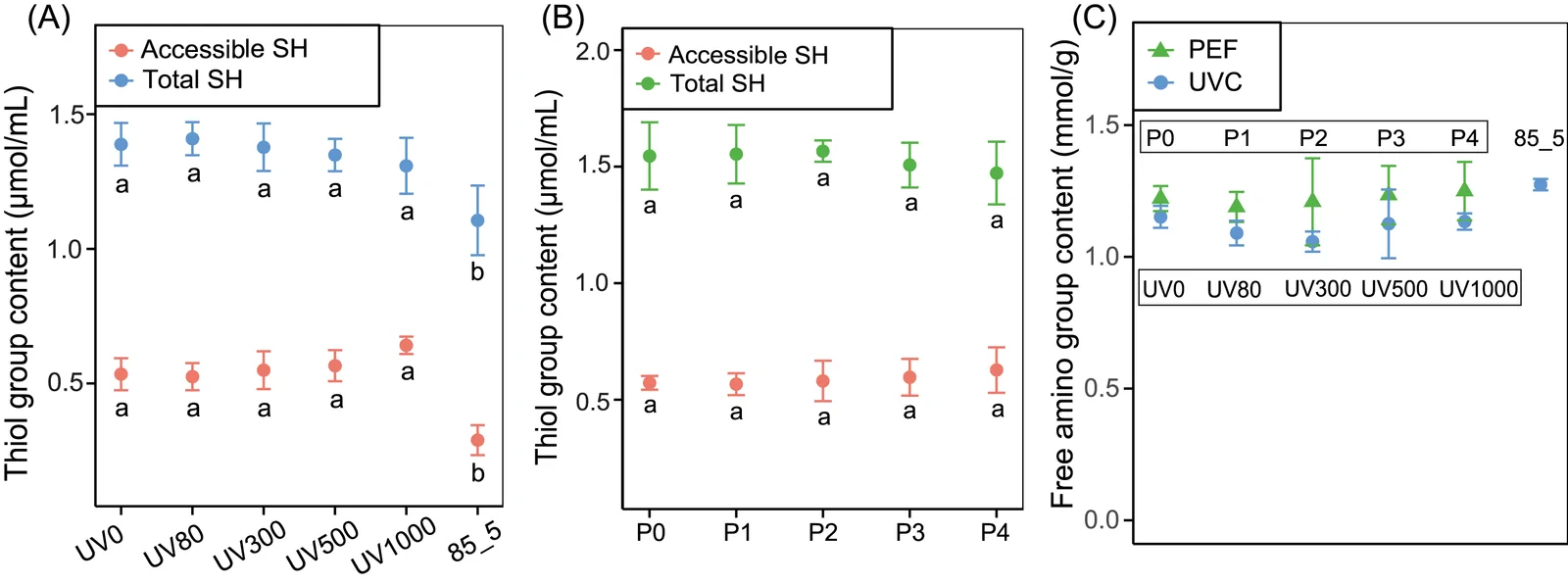
Thiol group content in skim milk after UVC, 85_5 (A), and nanoPEF (B) treatments; free amino group content in milk serum after UVC, nanoPEF and heating treatments (C). a-b means significant differences (*p* < 0.05). Comparisons between accessible SH and total SH were conducted separately. UV0-UV1000: UV0 is raw milk, UV80-UV1000 are milk irradiated from low to high intensity UVC; P0-P4: P0 is raw milk, P2-P4 are milk treated with low to high intensity nanoPEF; 85_5 is heated at 85 °C for 5 min. Data are presented as mean ± SD (n = 3 independent processing replicates). SH, thiol groups.

Besides the exposure of hydrophobic groups, another potential consequence of protein unfolding is the exposure of thiol (-SH) groups, which can further lead to the rearrangement of disulfide bonds. The accessible and total thiol group contents in skim milk after treatments are presented in Fig. 7. Both accessible and total thiol levels significantly decreased in the 85_5 sample (*p* < 0.05) as compared to the untreated sample. This suggests that more thiol groups were exposed and subsequently further oxidized and/or converted into intermolecular disulfide bonds, leading to the formation of aggregates that were partly removed by ultracentrifugation. In contrast, a slight, but non-significant, increase in accessible thiols and a decrease in total thiols were observed in UVC and nanoPEF samples. This indicates that only a very limited fraction of whey proteins adopts a loosened structure that exposes thiol groups, accompanied by little protein aggregation induced by disulfide bond rearrangement. However, these changes are negligible. This observation is consistent with the results obtained from SDS-PAGE (Fig. 5) and surface hydrophobicity (Fig. 6). Likewise, Hu et al. (2024) also reported that short-duration pulsed electric field treatment does not affect thiol content, with a significant decrease observed only when the treatment duration reached 8 ms or longer. Although disulfide bond cleavage and oxidation may occur with moderate to high UVC intensity (Fitzner et al., 2023), the conditions used in this study were apparently mild enough to prevent such reactions from happening.

#### 3.3.3 Free amino group contents

Several bands with molecular weight below 15 kDa were observed on the SDS-PAGE gel of heat-treated milk serum (Fig. 5). The amount of free amino groups was measured to investigate whether heating, UVC, or nanoPEF promoted peptide bond cleavage (Fig. 7C). No significant changes (p > 0.05) in the free amino group content were observed in milk serum after any of the treatments. This agrees with Bikaki et al. (2021), who also reported that peptide bond cleavage occurs only under extreme heat treatment conditions (220 °C).

The observations of these structural changes, combined with the results of native milk serum protein content and SDS-PAGE analysis, together show that intense heating (85 °C, 5 min) leads to the unfolding, denaturation, cross-linking, and aggregation of most milk serum proteins. With increasing UVC and nanoPEF treatment intensities, some non-significant changes occurred, showing very limited structural changes during both treatments.

### 3.4 Milk serum protein oxidation

**Fig. 8:**
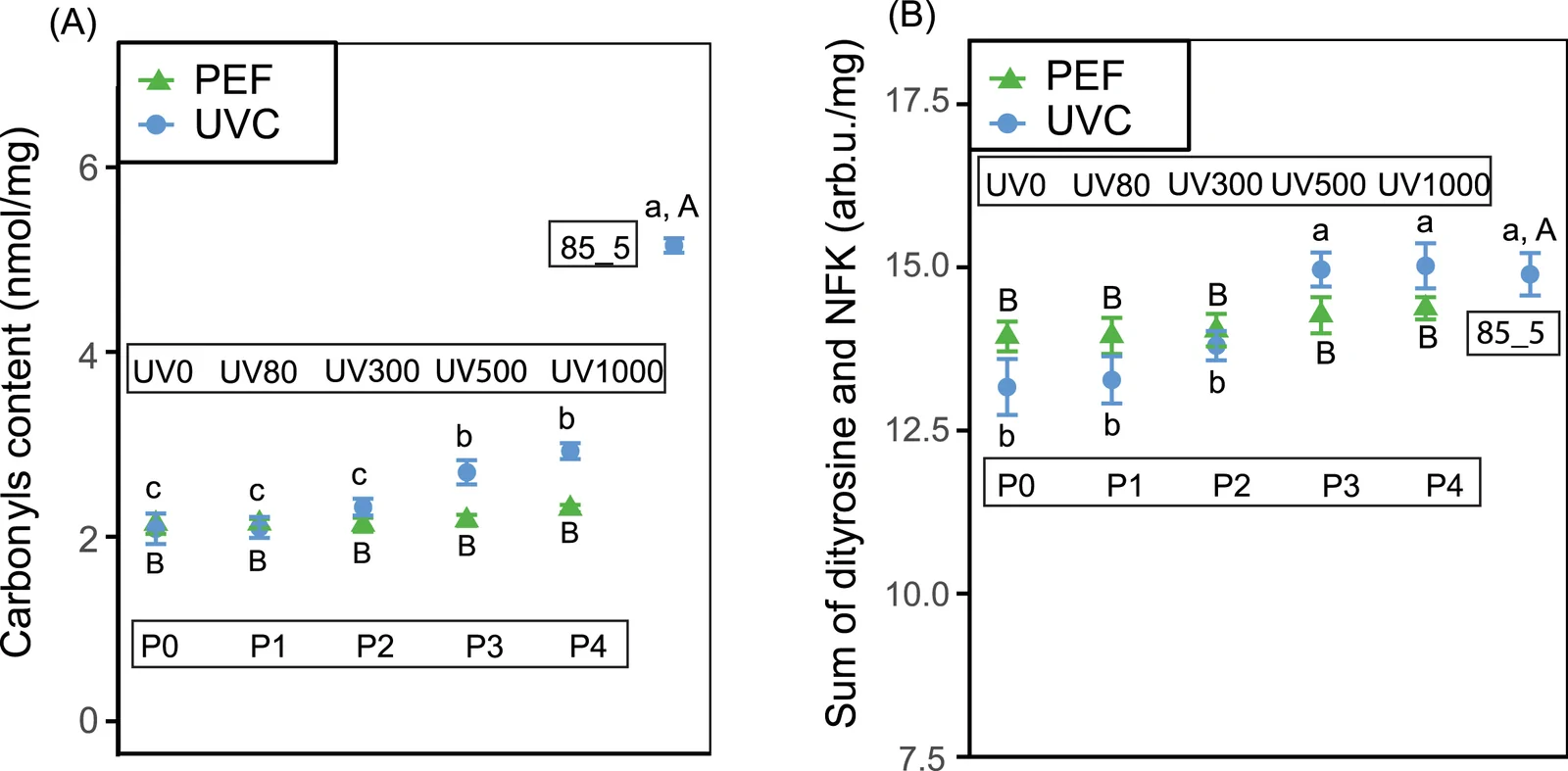
Protein-bound carbonyl content (A) and sum of dityrosine and NFK fluorescence (B) of UVC-, nanoPEF- and 85_5 treated milk serum. a-b and A-B indicate significant differences (p < 0.05) between UVC/85_5 and nanoPEF/85_5, respectively. UV0-UV1000: UV0 is raw milk, UV80-UV1000 are milk irradiated from low to high intensity UVC; P0-P4: P0 is raw milk, P2-P4 are milk treated with low to high intensity nanoPEF; 85_5 is heated at 85 °C for 5 min. Data are presented as mean ± SD (n = 3 independent processing replicates).

As mentioned earlier, the oxidation of sulfhydryl groups may contribute to disulfide bond cross-linking. Therefore, the degree of milk serum protein oxidation was assessed. Various amino acid residues may undergo oxidation, leading to the formation of carbonyls, N-formylkynurenine (NFK), and dityrosine. Therefore, these compounds are commonly used as markers to assess the extent of milk serum protein oxidation. As shown in Fig. 8A, after heating at 85°C for 5 min, the content of protein-bound carbonyls increased sharply, reaching levels approximately 2.5 times higher than those observed in the UVC- and nanoPEF-treated samples. When the UVC treatment intensity was increased to UV500, a significant increase (*p* < 0.05) in protein-bound carbonyl content was also observed. In contrast, no significant changes (*p* > 0.05) were detected in the nanoPEF-treated samples. The result of the UVC treatment, with slight increases upon extensive UVC treatment, is consistent with those reported by Fitzner et al. (2023), who observed a similar increase in protein-bound carbonyls after UVB irradiation of milk serum protein for 2 h. This increase may be related to direct photochemical oxidation of amino acid side chains, particularly those in aromatic and sulfur-containing residues. Aromatic amino acids (e.g., tryptophan, tyrosine, and phenylalanine) possess conjugated π-electron systems that strongly absorb UV radiation, thereby enhancing photooxidation and the formation of carbonyl-containing by-products (Cockell, 1998). Although UVC irradiation also triggered these changes, the effects were less pronounced than those caused by UVB and UVA (Pattison et al., 2012). Thus, direct photooxidation may have contributed to the protein aggregation observed in the high-dose UVC-treated samples. In contrast, no significant increase in the measured oxidation markers was observed after nanoPEF treatment under the conditions tested. Other researchers have found that significant protein oxidation occurs only under extremely high electric field strengths and/or prolonged exposure times. Hu et al. (2024) reported that exposing milk serum proteins to an electric field of 20 kV/cm for 8 ms (approximately 50 times higher than the conditions used in this study) resulted in an increase in carbonyl content by about 25%. In the 85_5 sample, a greater extent of carbonyls was observed. This increase may be associated with heat-induced oxidative reactions involving protein side chains (Milkovska-Stamenova et al., 2017).

Dityrosine and NFK are also recognized as markers of protein oxidation, representing the products of oxidative cross-linking of tyrosine residues and tryptophan oxidation, respectively. As shown in Fig. 8B, the levels of dityrosine and NFK increased significantly (p < 0.05) after UVC500 and UVC1000 treatments, resulting in a similar level to that found in the 85_5 sample. Similarly, Scheidegger et al. (2010) reported significant increases in dityrosine and NFK levels in both whole milk and skim milk following UV treatment. This is consistent with UVC-induced photochemical reactions involving tyrosine and tryptophan residues. Likewise, oxidation observed in the 85_5 sample was expected, as heating proteins at 80-90°C significantly accelerates protein oxidation and aggregation (Chen et al., 2005; C. Li et al., 2022). In contrast, the levels of dityrosine and NFK remained unchanged in the nanoPEF-treated samples, likely due to the absence of direct oxidation reactions under nanoPEF conditions, as discussed before.

The accumulation of photooxidation markers suggests that structural modifications occurred, which are likely to affect the functional behavior of milk serum proteins, including lactoferrin, immunoglobulins, and lactoperoxidase. These proteins rely on specific tertiary structures and intact amino acid residues for receptor binding and antimicrobial or immunomodulatory functions. Oxidative modification of aromatic and sulfur-containing residues may alter binding domains or metal-binding sites, potentially reducing antimicrobial and anti-inflammatory activity (Davies, 2016). Conversely, partial unfolding may enhance enzymatic susceptibility and promote the generation of bioactive peptides with immunomodulatory properties during digestion (Boutrou et al., 2015). However, their implications for biological activity require further experimental verification.

### 3.5 Milk serum protein proteome

**Fig. 9:**
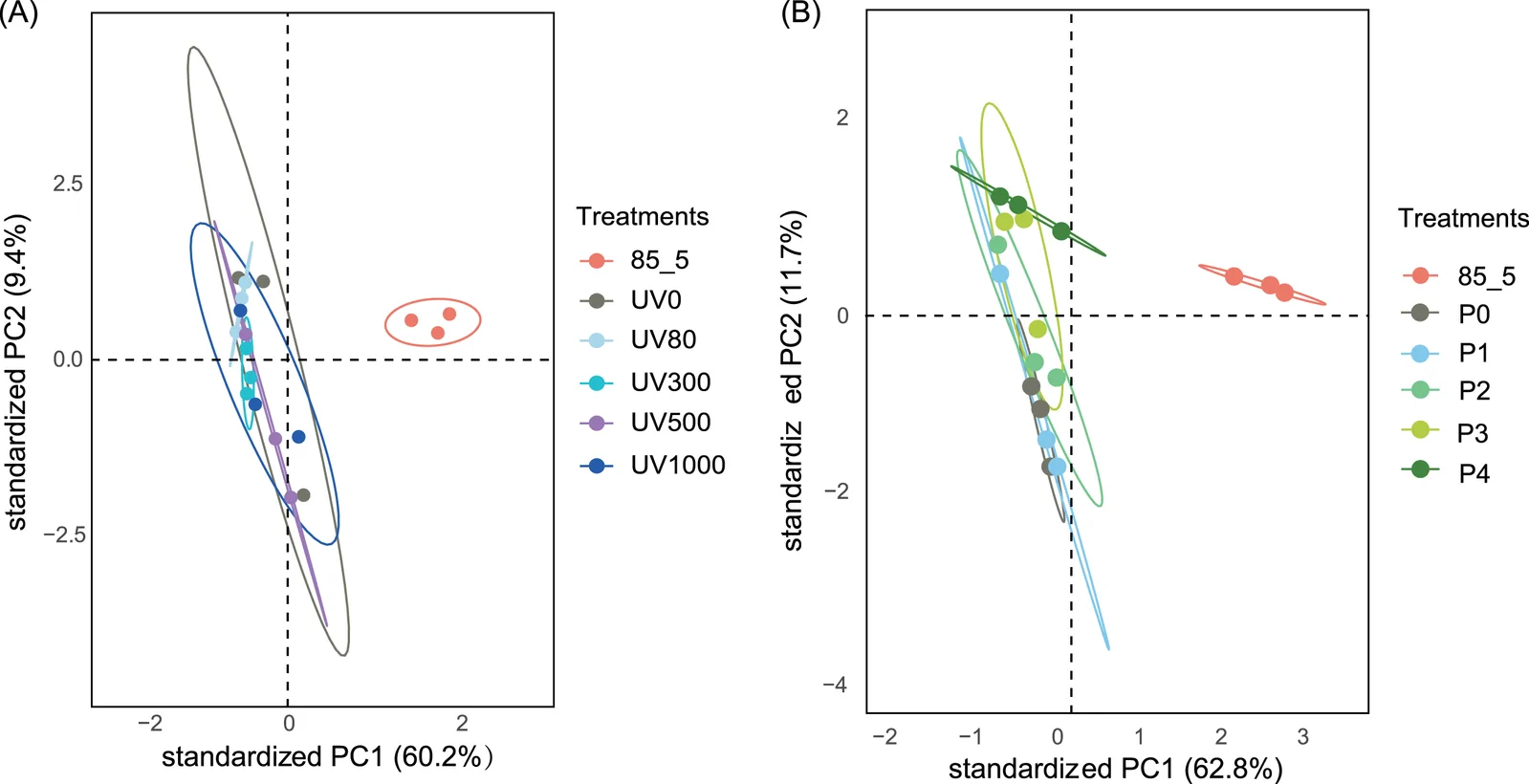
Principal Component Analysis (PCA) discriminated UVC and 85_5 (A); nanoPEF and 85_5 (B) treatment groups based on native protein profile. Each colored circle represents the 95% confidence ellipse of each group. Axis labels indicate the percentage of total variance explained by each principal component. UV0-UV1000: UV0 is raw milk, UV80-UV1000 are milk irradiated from low to high intensity UVC; P0-P4: P0 is raw milk, P2-P4 are milk treated with low to high intensity nanoPEF; 85_5 is heated at 85 °C for 5 min. Each point represents one independent processing replicate (n = 3 per treatment).

**Fig. 10:**
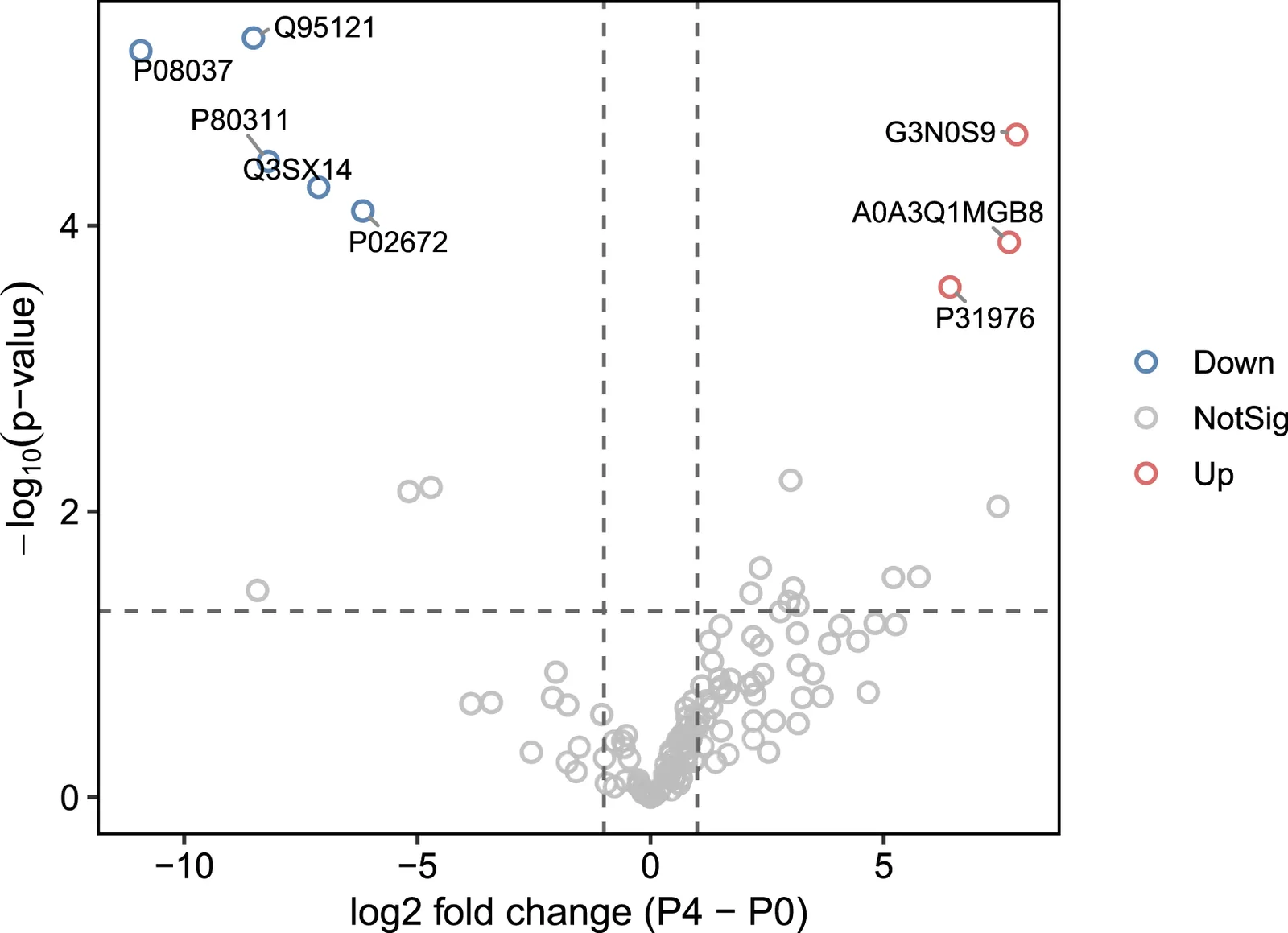
Volcano plot of differentially changed proteins between P0 and P4 samples. The x-axis represents log2 fold change (P4 vs. P0), and the y-axis represents −log10 (raw p-value). Proteins with |log2FC| ≥ 1 and FDR < 0.05 were considered significantly increased (red) or decreased (blue). n = 3 independent processing replicates per treatment.

To further investigate the effects of UVC, nanoPEF, and heat treatment on the milk serum protein profile, proteomic analysis was conducted using trypsin digestion and peptide identification and quantification by LC-MS/MS. Principal component analysis (PCA) was performed to assess differences in protein profiles among the samples. As shown in Fig. 9, the milk serum protein composition of the sample heated at 85 °C for 5 min was separated from all UVC-treated and nanoPEF-treated samples along PC1, which accounted for 60.2% and 62.8% of the total variation, respectively. No separation was observed between the UVC-treated groups and raw milk, indicating that UVC treatment did not markedly alter the overall composition of milk serum proteins under the conditions tested. The P4 sample, however, was separated from the untreated control along PC2, which explained 11.7% of the total variance. This suggests that within nanoPEF treatments, the duration of exposure to the electric field has a greater impact on milk serum protein composition than the associated temperature rise. Compared with P0, P4 treatment resulted in significant changes in 3 upregulated and 5 downregulated proteins (Fig. 10). Notably, these proteins are not major whey proteins, but are predominantly plasma-derived or cell-associated proteins. Importantly, Ezrin (P31976), a cytoskeleton–membrane linker protein, is commonly associated with the milk fat globule membrane (MFGM) (Dewettinck et al., 2008; Fehon et al., 2010). The observed decrease in Ezrin abundance may reflect changes in the recovery, solubility or detectability of membrane-associated proteins after nanoPEF treatment. However, the present data do not directly demonstrate changes in MFGM structural integrity. Overall, the milk serum protein profiles of both UVC- and nanoPEF-treated samples remained largely similar to that of raw milk.

**Fig. 11:**
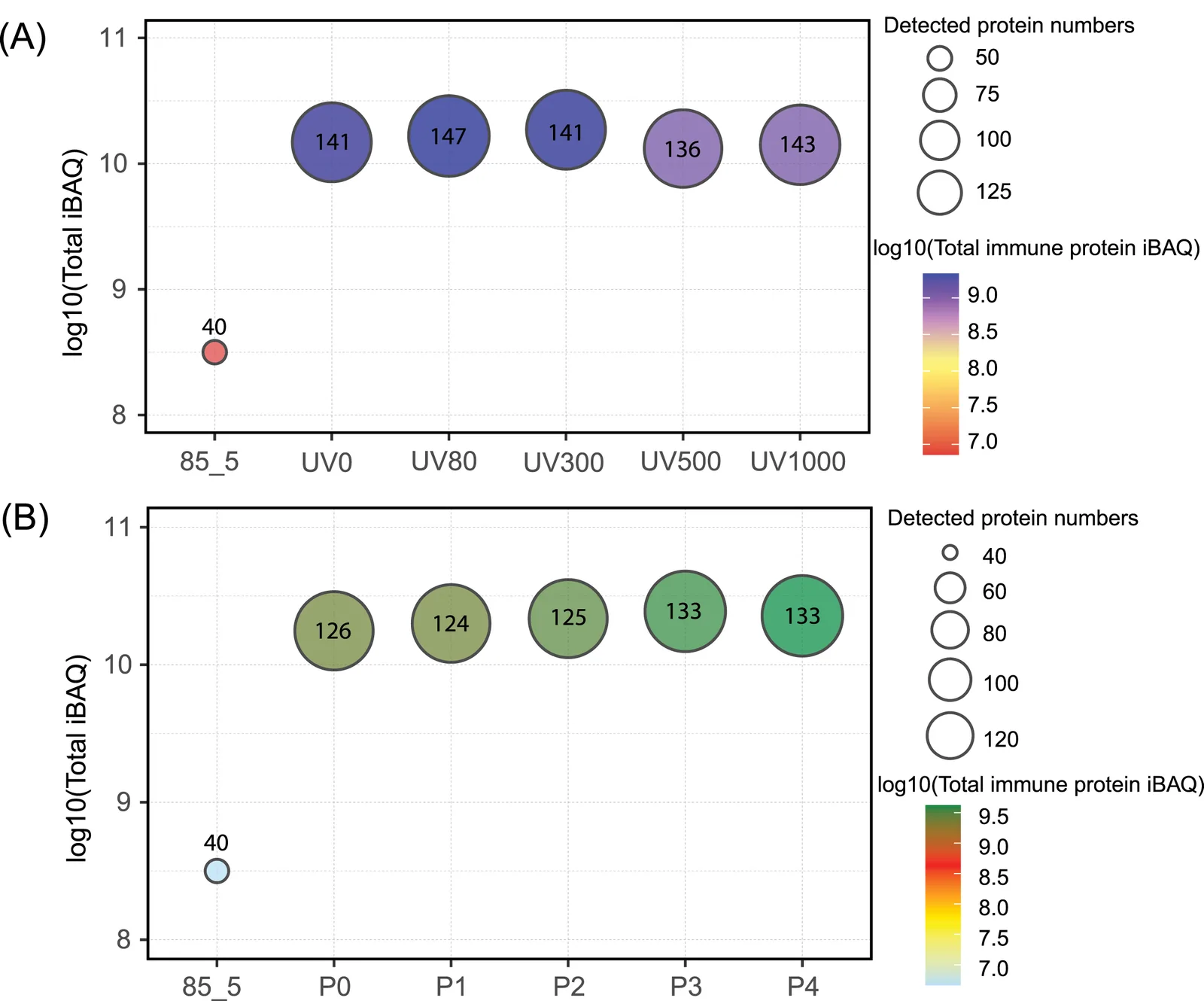
Bubble plots of milk serum samples treated by UVC/85_5 (A) and nanoPEF/85_5 (B). The vertical axis represents the logarithm of the sum of iBAQs for all detected proteins. The size of the bubble corresponds to the total number of proteins detected and is marked with numbers. The color of the bubble indicates the logarithm of the sum of iBAQs for all immune proteins. UV0-UV1000: UV0 is raw milk, UV80-UV1000 are milk irradiated from low to high intensity UVC; P0-P4: P0 is raw milk, P2-P4 are milk treated with low to high intensity nanoPEF; 85_5 is heated at 85 °C for 5 min n = 3 independent processing replicates per treatment.

The quantified milk serum proteins were visualized using a bubble chart, as shown in Fig. 11. More than 140 and 120 native milk serum proteins were detected in UVC- and nanoPEF-treated milk, respectively. In contrast, only around 40 milk serum proteins remained detectable in the heat-treated sample, as most were expected to be denatured, aggregated and removed during sample preparation. A similar treatment-dependent reduction in detectable milk serum proteins has been reported previously. In that study, 101 proteins were identified and quantified in raw milk serum, compared with 91 and 90 proteins after LTLT (63 °C) and HTST (72 °C) treatment, respectively, whereas only 56 proteins remained detectable after treatment at 85 °C (Y. Liu et al., 2020). These findings similarly indicate a greater loss of detectable serum proteins with increasing thermal intensity. In addition, the total abundance of milk serum proteins in both UVC- and nanoPEF-treated samples showed no significant difference compared to the untreated group, and remained markedly higher than in the heat-treated group. These findings are consistent with the results obtained from the DUMAS analysis (Fig. 4).

**Fig. S1:**
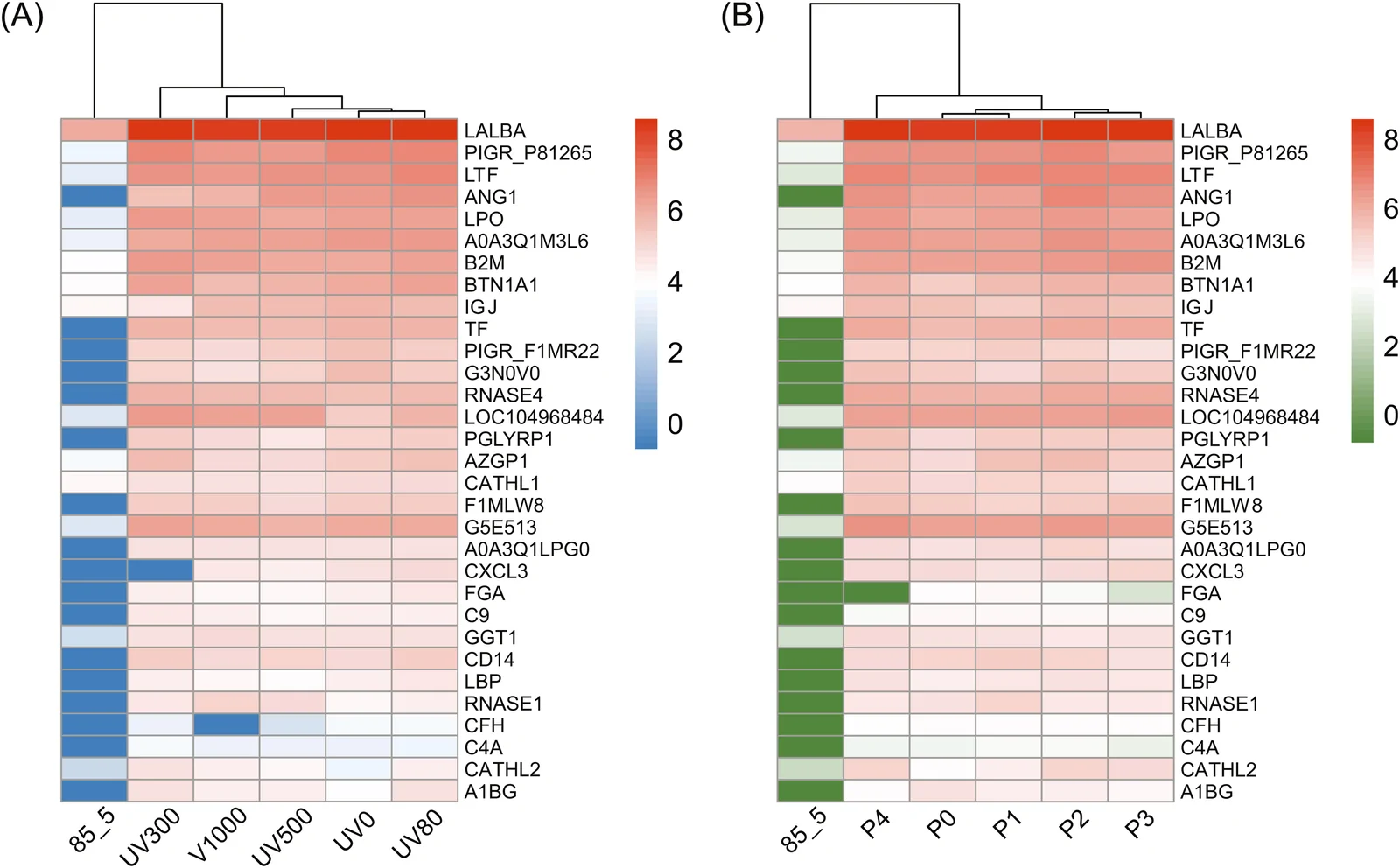
Hierarchical cluster analysis of immunologically-active milk serum proteins in UVC/85_5 (A) and nanoPEF/85_5 (B) samples based on the protein functions classified by gene ontology (GO)_Biological Process (BP) using the online tool Panther (https://www.pantherdb.org/). Proteins were named by their gene names. n = 3 independent processing replicates per treatment.

**Fig. S2:**
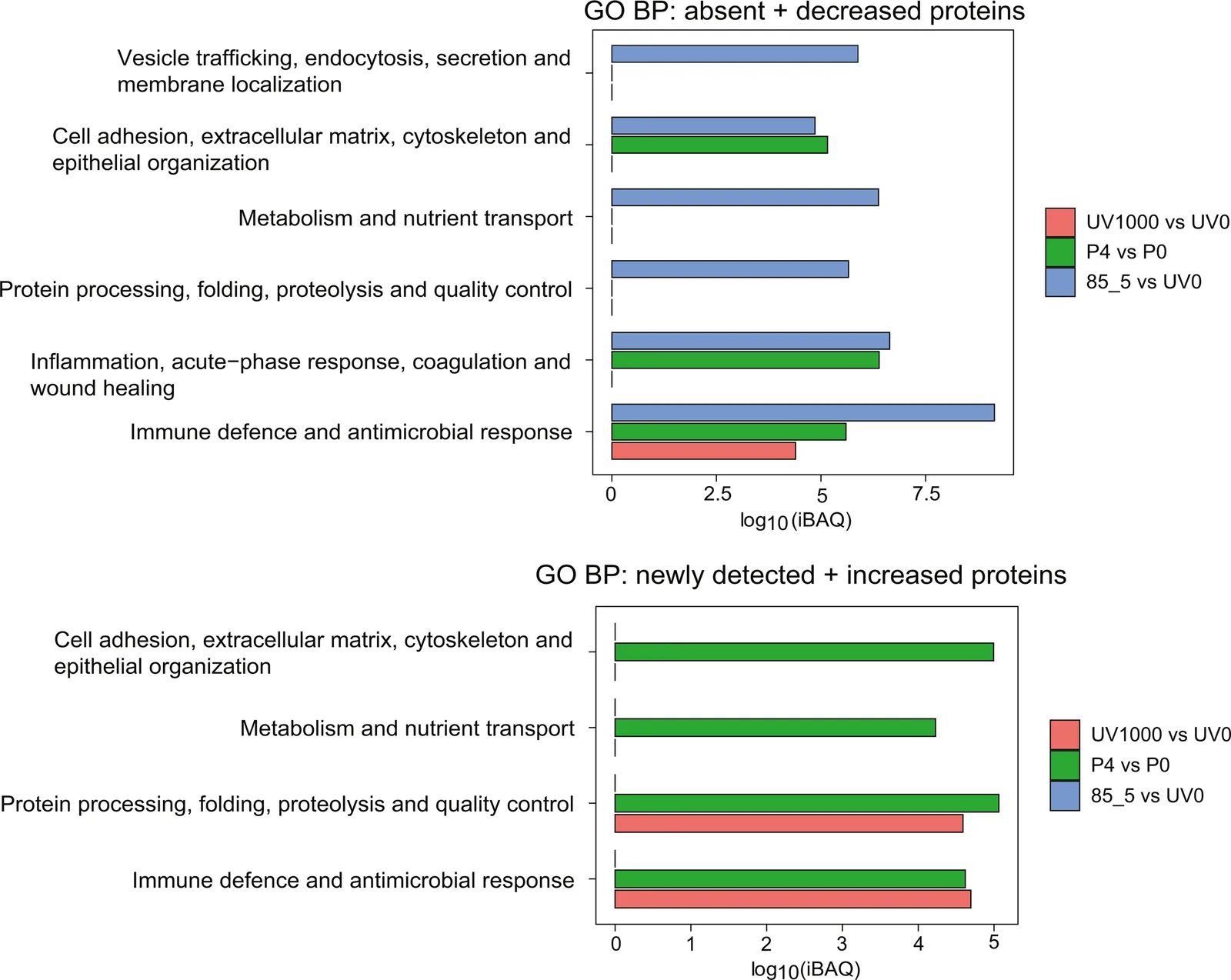
GO biological process classification of absent/decreased and newly detected/increased milk serum proteins after UVC, nanoPEF, and 85_5 treatments. Proteins were grouped into broad functional categories based on GO biological process annotations. n = 3 independent processing replicates per treatment.

To provide a broader functional overview of the detected proteins, treatment-affected proteins from the highest-intensity comparisons (UV1000 vs UV0, P4 vs P0, and 85_5 vs UV0) were grouped according to GO biological process annotations (Fig. S2). Across these comparisons, the largest changes were mainly associated with immune defence and antimicrobial response, particularly for the absent/decreased proteins after heat treatment (Table S2). Several of these proteins are associated with complement- and coagulation-related processes, while others, such as LBP and CD14, are involved in bacterial lipopolysaccharide recognition and innate immune signalling. Pathway-based proteomic studies of milk and whey have similarly highlighted complement/coagulation and other immune-related pathways as important functional networks in milk (Sun et al., 2020; L. Zhang et al., 2015). Therefore, immune-related proteins were further visualized in the bubble plot to compare their abundance changes across different processing conditions. Higher doses of UVC (UV500 and UV1000) led to a slight decrease in the abundance of immune-related proteins, as classified by the Panther database (Fig. S1), which is consistent with observations from the SDS-PAGE analysis (Fig. 5). This decrease should be interpreted as reduced abundance or detectability of these proteins in the prepared milk serum fraction, rather than direct evidence of functional loss. Possible explanations include partial denaturation or aggregation leading to reduced recovery during preparation of milk serum, or oxidation-related structural modifications affecting trypsin digestion, peptide recovery, or LC-MS/MS detection. However, these mechanisms were not directly tested in the present study. For nanoPEF-treated samples, a slight, though not statistically significant, increase in immune-related proteins was observed with increasing nanoPEF treatment intensity, which contrasts with the results shown by SDS-PAGE (Fig. 5). This discrepancy may be explained by nanoPEF-induced structural changes, such as slight protein unfolding, which could increase trypsin accessibility and thereby affect peptide detection by LC-MS/MS. However, further targeted analysis would be required to confirm this explanation.

Although the present study showed higher retention of residual native milk serum proteins and limited structural or oxidative changes after UVC and nanoPEF treatments compared with the high-intensity pasteurization, protein concentration and structural markers do not directly demonstrate preservation of biological function. Further studies of heat-sensitive bioactive proteins, such as lactoferrin, lactoperoxidase, and immunoglobulins, as well as overall antimicrobial activity, are required to determine whether the retained proteins also preserve their biological functions after processing.

### 3.6 Industrial relevance and practical implementation considerations

High-intensity pasteurization was used in this study as a reference for more extensive heat-induced protein denaturation and aggregation. In comparison, HTST treatment at 72 °C for 15 s is a relevant industrial benchmark because of its widespread application in milk pasteurization and may lead to more limited heat damage, although heat-sensitive bioactive proteins such as lactoferrin, immunoglobulins, lactoperoxidase, and xanthine oxidase may still be affected (Bogahawaththa and Vasiljevic, 2020; Wang et al., 2024). Therefore, future studies should include HTST and other thermal treatments with different intensities to more comprehensively compare UVC and nanoPEF with different thermal pasteurization conditions.

For practical implementation, microbiological and regulatory validation should also be considered. Targeted pathogen challenge tests or surrogate-based validation would be required before UVC or nanoPEF treatments could be considered equivalent to industrial pasteurization from a food safety perspective. In addition, regulatory and labelling requirements may differ between regions and intended applications. For example, UV-treated milk has been assessed as a novel food in the EU, and authorized products may require specific labelling such as “UV-treated milk”.

From an energy perspective, non-thermal processing is often considered attractive because microbial inactivation can be achieved without heating to pasteurization temperature and subsequently cooling it. However, the energy performance of these technologies is highly process- and equipment-dependent. For UVC treatment, the actual energy requirement depends on parameters such as lamp efficiency and target UVC dose. A recent review reported substantially lower energy usage for UV-C pasteurization than for conventional thermal pasteurization, indicating its potential advantage in reducing processing energy requirements (Grandsir et al., 2023). For nanoPEF, energy demand is affected by electric field strength, pulse and product parameters, and possible integration with mild preheating or heat recovery (Landi et al., 2025; Rodriguez-Gonzalez et al., 2015). A simulation study of fluid milk processing further showed that PEF does not necessarily provide an energy or environmental advantage over HTST, with its performance depends strongly on system configuration and operating conditions (Tomasula et al., 2013). Therefore, direct quantitative comparison across UVC, PEF, and thermal processing remains difficult because reported energy usage will depend on differences in equipment configuration, processing capacity, treatment intensity, and the system boundaries used for energy assessment.

The potential impact of oxidation on product quality should also be considered when evaluating the practical application of UVC treatment. In this study, high-dose UVC treatment increased protein oxidation markers. Previous studies have shown that light- or UV-induced oxidation in milk can promote the generation of volatile compounds associated with off-flavours during storage (Brothersen et al., 2016; Clarke et al., 2021; Matak et al., 2007). Therefore, the oxidation observed at high UVC doses may have implications for flavour, aroma, shelf-life, and consumer acceptance. Shelf-life was not evaluated in the present study and would require an experimental design covering biological variability in initial microbiota combined with dedicated refrigerated storage.

Overall, industrial implementation of UVC and nanoPEF for milk processing will require integrated evaluation of microbial safety, protein functionality, sensory quality, energy demand, and regulatory compliance.

## 4 Conclusion

Industrial-scale UVC irradiation (0.384-4.8 kJ/L) and nano-second pulsed electric field (nanoPEF, 29-182 kJ/L) treatments were successfully applied to milk and reduced naturally occurring microbial counts. Meanwhile, mild UVC and nanoPEF retained over 88% and 95% of native milk serum proteins, respectively, compared to only 56% in the milk heated at 85 °C for 5 min. In addition, no significant structural changes were observed in UVC or nanoPEF treatment, suggesting that these methods did not induce notable protein unfolding or aggregation. Although high-intensity UVC exposure (>2.4 kJ/L) led to a slight increase in protein oxidation products, this remained much lower compared to heat-treated samples.

Overall, this study demonstrates the potential of UVC and nanoPEF as non-thermal processing approaches for reducing microbial counts while better preserving milk serum protein structure compared with the high-intensity heat treatment used in this study. This study thereby provides a basis for future optimization of UVC and nanoPEF processing for industrial milk applications.

## CRediT authorship contribution statement

Siwei Li: Conceptualization, Methodology, Formal analysis, Investigation, Software, Writing – original draft, Writing – review & editing. Sjef Boeren: Formal analysis, Methodology, Software, Writing – review & editing. Betty C. A. *M. van* Esch: Conceptualization, Supervision, Writing – review & editing. Kasper A. Hettinga: Conceptualization, Software, Supervision, Validation, Writing – review & editing.

## Declaration of competing interests

The authors declare that they have no known competing financial interests or personal relationships that could have appeared to influence the work reported in this paper.

## References

[bib1] Abbring S., Xiong L., Diks M.A.P., Baars T., Garssen J., Hettinga K., van Esch B.C.A.M. (2020). Loss of allergy-protective capacity of raw cow's milk after heat treatment coincides with loss of immunologically active whey proteins. Food Funct..

[bib2] Araújo A., Barbosa C., Alves M.R., Romão A., Fernandes P. (2023). Implications of pulsed electric field pre-treatment on goat milk pasteurization. Foods.

[bib3] Bertsche D., Knipper P., Wetzel T. (2016). Experimental investigation on heat transfer in laminar, transitional and turbulent circular pipe flow. Int. J. Heat Mass Tran..

[bib4] Bikaki M., Shah R., Müller A., Kuhnert N. (2021). Heat induced hydrolytic cleavage of the peptide bond in dietary peptides and proteins in food processing. Food Chem..

[bib5] Bogahawaththa D., Vasiljevic T. (2020). Denaturation of selected bioactive whey proteins during pasteurization and their ability to modulate milk immunogenicity. J. Dairy Res..

[bib6] Boland M., Hill J. (2019). Milk Proteins: from Expression to Food.

[bib7] Boutrou R., Henry G., Sanchez-Rivera L. (2015). On the trail of milk bioactive peptides in human and animal intestinal tracts during digestion: a review. Dairy Sci. Technol..

[bib8] Brick T., Ege M., Boeren S., Böck A., von Mutius E., Vervoort J., Hettinga K. (2017). Effect of processing intensity on immunologically active bovine milk serum proteins. Nutrients.

[bib9] Brick T., Hettinga K., Kirchner B., Pfaffl M.W., Ege M.J. (2020). The beneficial effect of farm milk consumption on asthma, allergies, and infections: from meta-analysis of evidence to clinical trial. J. Allergy Clin. Immunol. Pract..

[bib10] Brothersen C., McMahon D.J., Legako J., Martini S. (2016). Comparison of milk oxidation by exposure to LED and fluorescent light. J. Dairy Sci..

[bib11] Chawla A., Lobacz A., Tarapata J., Zulewska J. (2021). UV light application as a mean for disinfection applied in the dairy industry. Appl. Sci..

[bib12] Chen W.L., Hwang M.T., Liau C.Y., Ho J.C., Hong K.C., Mao S.J.T. (2005). β-Lactoglobulin is a thermal marker in processed milk as studied by electrophoresis and circular dichroic spectra. J. Dairy Sci..

[bib13] Christen L., Lai C.T., Hartmann B., Hartmann P.E., Geddes D.T. (2013). The effect of UV-C pasteurization on bacteriostatic properties and immunological proteins of donor human milk. PLoS One.

[bib14] Clarke H.J., McCarthy W.P., O'Sullivan M.G., Kerry J.P., Kilcawley K.N. (2021). Oxidative quality of dairy powders: influencing factors and analysis. Foods.

[bib15] Cockell C.S. (1998). Ultraviolet radiation, evolution and the π-electron system. Biol. J. Linn. Soc..

[bib16] Davies M.J. (2016). Protein oxidation and peroxidation. Biochem. J..

[bib17] de Wit J.N., Kessel Th van (1996). Effects of ionic strength on the solubility of whey protein products. A colloid chemical approach. Food Hydrocoll..

[bib18] Dewettinck K., Rombaut R., Thienpont N., Le T.T., Messens K., Van Camp J. (2008). Nutritional and technological aspects of milk fat globule membrane material. Int. Dairy J..

[bib19] Digambar Sawale P., Subhash Patil P., Singh A., Raj Xavier J., Kumar P., Dutta D. (2024). Non-thermal techniques for microbiological safety, nutritional preservation, and enhanced efficiency in dairy processing. Functional Food Science.

[bib20] Fehon R.G., McClatchey A.I., Bretscher A. (2010). Organizing the cell cortex: the role of ERM proteins. Nat. Rev. Mol. Cell Biol..

[bib21] Feng X., Li C., Ullah N., Cao J., Lan Y., Ge W., Hackman R.M., Li Z., Chen L. (2015). Susceptibility of whey protein isolate to oxidation and changes in physicochemical, structural, and digestibility characteristics. J. Dairy Sci..

[bib22] Fiege J.L., Hirt B., Gräf V., Nöbel S., Martin D., Fritsche J., Schrader K., Stahl M. (2022). Uridine as a non-toxic actinometer for UV-C treatment: influence of temperature and concentration. Heliyon.

[bib23] Fitzner L., Kühl T., Hasler M., Imhof D., Schwarz K., Keppler J.K. (2023). Modification and oxidative degradation of β-lactoglobulin by UVB irradiation. Food Chem..

[bib24] Grandsir C., Falagán N., Alamar M.C. (2023). Application of novel technologies to reach net-zero greenhouse gas emissions in the fresh pasteurised milk supply chain: a review. Int. J. Dairy Technol..

[bib25] Hettinga K., van Valenberg H., de Vries S., Boeren S., van Hooijdonk T., van Arendonk J., Vervoort J. (2011). The host defense proteome of human and bovine milk. PLoS One.

[bib26] Hu X., Wang H., Hu Y., Tu Z. (2024). Insight into the effects of pulsed electric field on the structure, aggregation characteristics and functional properties of whey proteins. Food Hydrocoll..

[bib27] Kontopodi E., Stahl B., van Goudoever J.B., Boeren S., Timmermans R.A.H., den Besten H.M.W., Van Elburg R.M., Hettinga K. (2022). Effects of high-pressure processing, UV-C irradiation and thermoultrasonication on donor human milk safety and quality. Front. Pediatr..

[bib28] Lam R.S.H., Nickerson M.T. (2015). The effect of pH and temperature pre-treatments on the physicochemical and emulsifying properties of whey protein isolate. LWT Food Sci. Technol. (Lebensm.-Wiss.-Technol.).

[bib29] Landi G., Benedetti M., Sforzini M., Eslami E., Pataro G. (2025). Comparative analysis of cost, energy efficiency, and environmental impact of pulsed electric fields and conventional thermal treatment with integrated heat recovery for fruit juice pasteurization. Foods.

[bib30] Law A.J.R., Leaver J. (2000). Effect of pH on the thermal denaturation of whey proteins in milk. J. Agric. Food Chem..

[bib31] Lee G., Han B., Choi H., Kang S., Baick S., Lee D.-U. (2015). Inactivation of Escherichia coli, Saccharomyces cerevisiae, and Lactobacillus brevis in low-fat milk by pulsed electric field treatment: a pilot-scale study. Korean J. Food Sci. Anim. Resour..

[bib32] Li C., Nielsen S.B., Engholm-Keller K., Lund M.N. (2022). Oxidation of whey proteins during thermal treatment characterized by a site-specific LC–MS/MS-Based proteomic approach. J. Agric. Food Chem..

[bib33] Li Z., Liu D., Xu S., Zhang W., Hemar Y., Regenstein J.M., Zhou P. (2021). Effects of pasteurization, microfiltration, and ultraviolet-c treatments on microorganisms and bioactive proteins in bovine skim milk. Food Biosci..

[bib34] Lin T., Meletharayil G., Kapoor R., Abbaspourrad A. (2021). Bioactives in bovine milk: chemistry, technology, and applications. Nutr. Rev..

[bib35] Liu H., May K. (2012). Disulfide bond structures of IgG molecules. mAbs.

[bib36] Liu L., Lin S., Ma S., Sun Y., Li X., Liang S. (2022). A comparative analysis of lipid digestion in human milk and infant formulas based on simulated in vitro infant gastrointestinal digestion. Foods.

[bib37] Liu Y., Shuang J., Hettinga K., Zhang L., Liu X., Zhou P. (2024). Quality attributes of whole milk powder processed using ultrasonication and UV-C radiation compared with equivalent thermal treatment. Int. Dairy J..

[bib38] Liu Y., Xiong L., Kontopodi E., Boeren S., Zhang L., Zhou P., Hettinga K. (2020). Changes in the milk serum proteome after thermal and non-thermal treatment. Innov. Food Sci. Emerg. Technol..

[bib39] Liu Y., Zhang W., Han B., Zhang L., Zhou P. (2020). Changes in bioactive milk serum proteins during milk powder processing. Food Chem..

[bib40] Loss G., Apprich S., Waser M., Kneifel W., Genuneit J., Büchele G., Weber J., Sozanska B., Danielewicz H., Horak E., Joost van Neerven R.J., Heederik D., Lorenzen P.C., von Mutius E., Braun-Fahrländer C., Boznanski A., Cookson W., Cullinan P., Debinśka A. (2011). The protective effect of farm milk consumption on childhood asthma and atopy: the GABRIELA study. J. Allergy Clin. Immunol..

[bib41] Lowe E.K., Anema S.G., Bienvenue A., Boland M.J., Creamer L.K., Jiménez-Flores R. (2004). Heat-induced redistribution of disulfide bonds in milk proteins. 2. Disulfide bonding patterns between bovine β-Lactoglobulin and κ-Casein. J. Agric. Food Chem..

[bib42] Makararpong D., Tantayanon S., Gowanit C., Jareonsawat J., Samgnamnim S., Wataradee S., Hogeveen H., Inchaisri C. (2024). Enhancing raw bovine milk quality using Ultraviolet-C (UV-C) irradiation: a microbial and lipid peroxidation study. Food Sci. Anim. Resour..

[bib43] Manzo C., Nicolai M.A., Pizzano R. (2015). Thermal markers arising from changes in the protein component of milk. Food Control.

[bib44] Matak K.E., Sumner S.S., Duncan S.E., Hovingh E., Worobo R.W., Hackney C.R., Pierson M.D. (2007). Effects of ultraviolet irradiation on chemical and sensory properties of goat milk. J. Dairy Sci..

[bib45] Milkovska-Stamenova S., Mnatsakanyan R., Hoffmann R. (2017). Protein carbonylation sites in bovine raw milk and processed milk products. Food Chem..

[bib46] Morand M., Guyomarc’h F., Pezennec S., Famelart M.-H. (2011). On how κ-casein affects the interactions between the heat-induced whey protein/κ-casein complexes and the casein micelles during the acid gelation of skim milk. Int. Dairy J..

[bib47] Novickij V., Zinkevičienė A., Stanevičienė R., Gruškienė R., Servienė E., Vepštaitė-Monstavičė I., Krivorotova T., Lastauskienė E., Sereikaitė J., Girkontaitė I., Novickij J. (2018). Inactivation of Escherichia coli using nanosecond electric fields and nisin nanoparticles: a kinetics study. Front. Microbiol..

[bib48] Owusu-Darko R., Allam M., de Oliveira S.D., Ferreira C.A.S., Grover S., Mtshali S., Ismail A., Mallappa R.H., Tabit F., Buys E.M. (2019). Genome sequences of Bacillus sporothermodurans strains isolated from ultra-high-temperature milk. Microbiol. Resour. Announc..

[bib49] Pattison D.I., Rahmanto A.S., Davies M.J. (2012). Photo-oxidation of proteins. Photochem. Photobiol. Sci..

[bib50] Perkin M.R., Strachan D.P. (2006). Which aspects of the farming lifestyle explain the inverse association with childhood allergy?. J. Allergy Clin. Immunol..

[bib51] Petersen R.C. (2017).

[bib52] Ranieri M.L., Huck J.R., Sonnen M., Barbano D.M., Boor K.J. (2009). High temperature, short time pasteurization temperatures inversely affect bacterial numbers during refrigerated storage of pasteurized fluid milk. J. Dairy Sci..

[bib53] Rodriguez-Gonzalez O., Buckow R., Koutchma T., Balasubramaniam V.M. (2015). Energy requirements for alternative food processing technologies—principles, assumptions, and evaluation of efficiency. Compr. Rev. Food Sci. Food Saf..

[bib54] Ruiz-Fernández A.R., Campos L., Gutierrez-Maldonado S.E., Núñez G., Villanelo F., Perez-Acle T. (2022). Nanosecond pulsed electric field (nsPEF): opening the biotechnological Pandora's box. Int. J. Mol. Sci..

[bib55] Scheidegger D., Larsen G., Kivatinitz S.C. (2016). Oxidative consequences of UV irradiation on isolated milk proteins: effects of hydrogen peroxide and bivalent metal ions. Int. Dairy J..

[bib56] Scheidegger D., Pecora R.P., Radici P.M., Kivatinitz S.C. (2010). Protein oxidative changes in whole and skim milk after ultraviolet or fluorescent light exposure. J. Dairy Sci..

[bib57] Singh P.K., Huppertz T., Boland M., Singh H. (2020). Milk Proteins.

[bib58] Smelt J.P.P.M., Brul S. (2014). Thermal inactivation of microorganisms. Crit. Rev. Food Sci. Nutr..

[bib59] Sozańska B. (2019). Raw cow's milk and its protective effect on allergies and asthma. Nutrients.

[bib60] Sui Q., Roginski H., Williams R.P.W., Versteeg C., Wan J. (2011). Effect of pulsed electric field and thermal treatment on the physicochemical and functional properties of whey protein isolate. Int. Dairy J..

[bib61] Sun Y., Wang C., Sun X., Guo M. (2020). Proteomic analysis of whey proteins in the colostrum and mature milk of Xinong Saanen goats. J. Dairy Sci..

[bib62] Tomasula P.M., Yee W.C.F., McAloon A.J., Nutter D.W., Bonnaillie L.M. (2013). Computer simulation of energy use, greenhouse gas emissions, and process economics of the fluid milk process1. J. Dairy Sci..

[bib63] Vashisht P., Singh L., Mahanta S., Verma D., Sharma S., Saini G.S., Sharma A., Chowdhury B., Awasti N., Gaurav, Mahanta S., Chauhan D.S. (2024). Pulsed electric field processing in the dairy sector: a review of applications, quality impact and implementation challenges. Int. J. Food Sci. Technol..

[bib64] Wang Y., Xiao R., Liu S., Wang P., Zhu Y., Niu T., Chen H. (2024). The impact of thermal treatment intensity on proteins, fatty acids, Macro/micro-nutrients, flavor, and heating markers of milk—A comprehensive review. Int. J. Mol. Sci..

[bib65] Waser M., Michels K.B., Bieli C., Flöistrup H., Pershagen G., Von Mutius E., Ege M., Riedler J., Schram-Bijkerk D., Brunekreef B., Van Hage M., Lauener R., Braun-Fahrländer C., team, the P. S. (2007). Inverse association of farm milk consumption with asthma and allergy in rural and suburban populations across Europe. Clin. Exp. Allergy.

[bib66] Wiśniewski J.R., Zougman A., Nagaraj N., Mann M. (2009). Universal sample preparation method for proteome analysis. Nat. Methods.

[bib67] Yan Z., Yin L., Hao C., Liu K., Qiu J. (2021). Synergistic effect of pulsed electric fields and temperature on the inactivation of microorganisms. AMB Express.

[bib68] Yüksel Z., Erdem Y.K. (2005). The influence of main milk components on the hydrophobic interactions of milk protein system in the course of heat treatment. J. Food Eng..

[bib69] Zhang J., Ghasemi N., Zare F., Duley J.A., Cowley D.M., Shaw P.N., Koorts P., Bansal N. (2023). Nanosecond pulsed electric field treatment of human milk: effects on microbiological inactivation, whey proteome and bioactive protein. Food Chem..

[bib70] Zhang L., Boeren S., Hageman J.A., van Hooijdonk T., Vervoort J., Hettinga K. (2015). Bovine milk proteome in the first 9 days: protein interactions in maturation of the immune and digestive system of the newborn. PLoS One.

[bib71] Zhao Z., Engholm-Keller K., Poojary M.M., Boelt S.G., Rogowska-Wrzesinska A., Skibsted L.H., Davies M.J., Lund M.N. (2020). Generation of aggregates of α-Lactalbumin by UV-B light exposure. J. Agric. Food Chem..

