# Supplementary material for: Pilot-scale ultraviolet-C and nano-second pulsed electric field processing of milk: Microbial inactivation and preservation of serum proteins
Source: Curr Res Food Sci. 2026 Aug 26;13:101546. doi: 10.1016/j.crfs.2026.101546 (PMC13545443; doi:10.1016/j.crfs.2026.101546)
Supplement: Supplementary file 1 [file mmc1.docx]

**Table S1.** Relative band intensities of milk serum proteins* in the milk serum on the SDS-PAGE gel under reducing (A) and non-reducing (B) conditions. LF: lactoferrin, LPO: lactoperoxidase, BSA: bovine serum albumin, Ig-HC: IgG-heavy chain, β-Lg: β-lactoglobulin, α-la: α-lactalbumin.

(A)

|  | **UV0** | **UV**  **80** | **UV**  **300** | **UV**  **500** | **UV**  **1000** | **85_5** | **P0** | **P1** | **P2** | **P3** | **P4** |
| --- | --- | --- | --- | --- | --- | --- | --- | --- | --- | --- | --- |
| **LF** | 100% | 95% | 90% | 89% | 70% | ND | 100% | 74% | 70% | 48% | 30% |
| **LPO** | 100% | 103% | 97% | 90% | 76% | ND | 100% | 76% | 72% | 57% | 43% |
| **BSA** | 100% | 101% | 97% | 95% | 79% | 23% | 100% | 72% | 68% | 71% | 57% |
| **Ig-HC** | 100% | 107% | 92% | 90% | 71% | ND | 100% | 73% | 66% | 61% | 54% |
| **β-Lg** | 100% | 101% | 97% | 98% | 84% | 59% | 100% | 86% | 84% | 93% | 88% |
| **α-La** | 100% | 102% | 95% | 94% | 74% | 82% | 100% | 81% | 81% | 93% | 83% |

* Percentages represent the protein band intensity relative to that of the untreated milk serum proteins (set as 100%). ND indicates not detected.

(B)

|  | **UV0** | **UV**  **80** | **UV**  **300** | **UV**  **500** | **UV**  **1000** | **85_5** | | **P0** | **P1** | **P2** | **P3** | **P4** |
| --- | --- | --- | --- | --- | --- | --- | --- | --- | --- | --- | --- | --- |
| **LF** | 100% | 86% | 83% | 72% | 55% | | ND | 100% | 68% | 61% | 52% | 43% |
| **LPO** | 100% | 114% | 78% | 67% | 22% | | ND | 100% | 67% | 59% | 44% | 35% |
| **BSA** | 100% | 101% | 95% | 88% | 53% | | 26% | 100% | 71% | 64% | 80% | 51% |
| **Ig-HC** | 100% | 104% | 101% | 98% | 95% | | ND | 100% | 44% | 43% | 32% | 30% |
| **β-Lg** | 100% | 102% | 102% | 94% | 84% | | 53% | 100% | 85% | 81% | 87% | 76% |
| **α-La** | 100% | 106% | 107% | 109% | 84% | | 88% | 100% | 66% | 68% | 88% | 69% |

* Percentages represent the protein band intensity relative to that of the untreated milk serum proteins (set as 100%). ND indicates not detected.
