# Supplementary material for: Pilot-scale ultraviolet-C and nano-second pulsed electric field processing of milk: Microbial inactivation and preservation of serum proteins
Source: Curr Res Food Sci. 2026 Aug 26;13:101546. doi: 10.1016/j.crfs.2026.101546 (PMC13545443; doi:10.1016/j.crfs.2026.101546)
Supplement: Supplementary file 2 [file mmc2.docx]

**Table S2.** Treatment-affected proteins classified under “immune defence and antimicrobial response” based on GO biological process annotations and their suggested biological pathway/functional context.

| **Comparison** | **Change** | **Protein name** | **Gene** | **Protein ID** | **Suggested biological pathway / functional context** |
| --- | --- | --- | --- | --- | --- |
| UV1000_vs_UV0 | Disappeared | Complement factor H | CFH | Q28085 | Complement system / complement regulation |
| UV1000_vs_UV0 | Appeared | Leukocyte elastase inhibitor | SERPINB1 | Q1JPB0 | Inflammatory regulation / innate immune response |
| P4_vs_P0 | Disappeared | Peptidyl-prolyl cis-trans isomerase B | PPIB | P80311 | Neutrophil chemotaxis / immune-cell recruitment |
| P4_vs_P0 | Disappeared | Fibrinogen alpha chain | FGA | P02672 | Complement/coagulation-related processes; coagulation and fibrinolysis |
| P4_vs_P0 | Appeared | Haptoglobin | HP | G3X6K8 | Acute-phase response / antibacterial defence |
| H85_vs_UV0 | Disappeared | Angiogenin-1 | ANG1 | P10152 | Antimicrobial humoral defence / innate immunity |
| H85_vs_UV0 | Disappeared | Serotransferrin | TF | Q29443 | Nutritional immunity / antibacterial defence |
| H85_vs_UV0 | Disappeared | Ribonuclease 4 | RNASE4 | Q58DP6 | Antibacterial humoral defence |
| H85_vs_UV0 | Disappeared | Polymeric immunoglobulin receptor | PIGR | F1MR22 | Mucosal immunity / immunoglobulin transport |
| H85_vs_UV0 | Disappeared | Peptidoglycan recognition protein 1 | PGLYRP1 | Q8SPP7 | Pattern recognition / antibacterial innate immunity |
| H85_vs_UV0 | Disappeared | Monocyte differentiation antigen CD14 | CD14 | Q95122 | LPS recognition / TLR4-mediated innate immune signalling |
| H85_vs_UV0 | Disappeared | Uncharacterized protein | — | A0A3Q1LPG0 | Classical complement pathway / antibacterial humoral defence |
| H85_vs_UV0 | Disappeared | C-X-C motif chemokine | CXCL3 | F1MD23 | Chemokine signalling / neutrophil recruitment |
| H85_vs_UV0 | Disappeared | Lipopolysaccharide-binding protein | LBP | A0A3Q1LKB0 | LPS recognition / Gram-negative bacterial defence |
| H85_vs_UV0 | Disappeared | Leucine-rich alpha-2-glycoprotein 1 | LRG1 | F6RMV5 | Immune response / TGF-beta signalling |
| H85_vs_UV0 | Disappeared | Ribonuclease pancreatic | RNASE1 | P61823 | Antibacterial defence |
| H85_vs_UV0 | Disappeared | Complement factor B | CFB | A0A3Q1LRP5 | Alternative complement pathway |
| H85_vs_UV0 | Disappeared | Ectonucleotide pyrophosphatase/phosphodiesterase family member 3 | ENPP3 | P15396 | Basophil/mast-cell immune regulation |
| H85_vs_UV0 | Disappeared | Complement component C9 | C9 | Q3MHN2 | Complement cascade / terminal complement (membrane attack complex) |
| H85_vs_UV0 | Disappeared | Fibrinogen alpha chain | FGA | P02672 | Complement/coagulation-related processes; coagulation and fibrinolysis |
| H85_vs_UV0 | Disappeared | Fibrinogen beta chain | FGB | P02676 | Complement/coagulation-related processes; fibrin clot formation |
| H85_vs_UV0 | Disappeared | Complement factor H | CFH | Q28085 | Complement system / complement regulation |
| H85_vs_UV0 | Disappeared | Cadherin-1 | CDH1 | Q6R8F2 | Epithelial barrier / cell-cell adhesion (source category: immune defence) |
| H85_vs_UV0 | Significantly decreased | Lactoperoxidase | LPO | P80025 | Lactoperoxidase antimicrobial system / antibacterial humoral defence |
| H85_vs_UV0 | Significantly decreased | Alpha-lactalbumin | LALBA | P00711 | Antibacterial defence |
| H85_vs_UV0 | Significantly decreased | Butyrophilin subfamily 1 member A1 | BTN1A1 | P18892 | T-cell / cytokine-related immune regulation |
| H85_vs_UV0 | Significantly decreased | Platelet glycoprotein 4 | CD36 | P26201 | Bacterial-lipopeptide sensing / inflammasome-related signalling |
| H85_vs_UV0 | Significantly decreased | Clusterin | CLU | P17697 | Immune-complex clearance / NF-kappaB-related immune regulation |
| H85_vs_UV0 | Significantly decreased | Zinc-alpha-2-glycoprotein | AZGP1 | Q3ZCH5 | Antigen presentation / T-cell-mediated immunity |
| H85_vs_UV0 | Significantly decreased | Ig-like domain-containing protein | LOC104968484 | A0A3Q1MUR2 | Adaptive / immunoglobulin-mediated immune response |

Proteins were classified under “immune defence and antimicrobial response” based on GO biological process annotations. The related biological process/functional context was summarized from these annotations and relevant literature; no pathway enrichment analysis was performed.
